# A Marine Stem-Tetrapod from the Devonian of Western North America

**DOI:** 10.1371/journal.pone.0033683

**Published:** 2012-03-20

**Authors:** Brian Swartz

**Affiliations:** Department of Integrative Biology and Museum of Paleontology, University of California, Berkeley, California, United States of America; Raymond M. Alf Museum of Paleontology, United States of America

## Abstract

The origin of terrestrial vertebrates represents one of the major evolutionary and ecological transformations in the history of life, and the established timing and environment of this transition has recently come under scrutiny. The discovery and description of a well-preserved fossil sarcopterygian (fleshy-limbed vertebrate) from the Middle Devonian of Nevada helps to refine and question aspects of the temporal and anatomical framework that underpins the tetrapod condition. This new taxon, *Tinirau clackae*, demonstrates that substantial parallelism pervaded the early history of stem-tetrapods, raises additional questions about when digited sarcopterygians first evolved, and further documents that incipient stages of the terrestrial appendicular condition began when sarcopterygians still retained their median fins and occupied aquatic habitats.

## Introduction

The origin and early evolution of tetrapodomorphs (total-group tetrapods) has been firmly established by numerous studies over the last two decades [1]–[7]. However, knowledge of the interrelationships among fish-like ‘osteolepiform’-grade taxa and the earliest elpistostegalians has remained elusive [8]–[11]. Phylogenetic analyses have reinforced hypotheses of ‘osteolepiform’ paraphyly and parallelism among Devonian stem-tetrapods, but lack of robust statistical support for particular topologies has limited our knowledge of branching and divergence in these early lineages [8], [12]. Few studies recover support for larger clades within the ‘osteolepidids’ [13], and several establish the close relationship of tristichopterids and elpistostegalians with a robust sister relationship between *Panderichthys* and early digited forms [3], [12], [14]. However, no new taxa so far known document the assembly of traits leading from tristichopterids to elpistostegalians.

The discovery of a new stem-tetrapod from the Middle Devonian of western North America helps to fill this gap and provides a stronger phylogenetic backbone upon which future studies can build. The new material includes several specimens from marine sediments and represents an animal with numerous elpistostegalian apomorphies, yet also many symplesiomorphies, suggesting that early tetrapodomorph features have a more crownward distribution than previously considered. This mélange of characters extends ancestral tetrapodomorph traits across the early history of the first digited forms, and as part of a phylogenetic hypothesis speaks to the length of current ghost ranges implied by the early Middle Devonian Zachełmie (Polish) trackways [15]. However, considering the late Middle Devonian age of this taxon, its congruence with the stratophylogenetic records of other stem-tetrapods, and the phylogenetic distribution of locomotor gaits among crown-group sarcopterygians, questions about when the first digited sarcopterygians first evolved should be considered a more open question than what a strict reading of the trace fossil record might imply.

### Geological Framework

**Figure 1:**
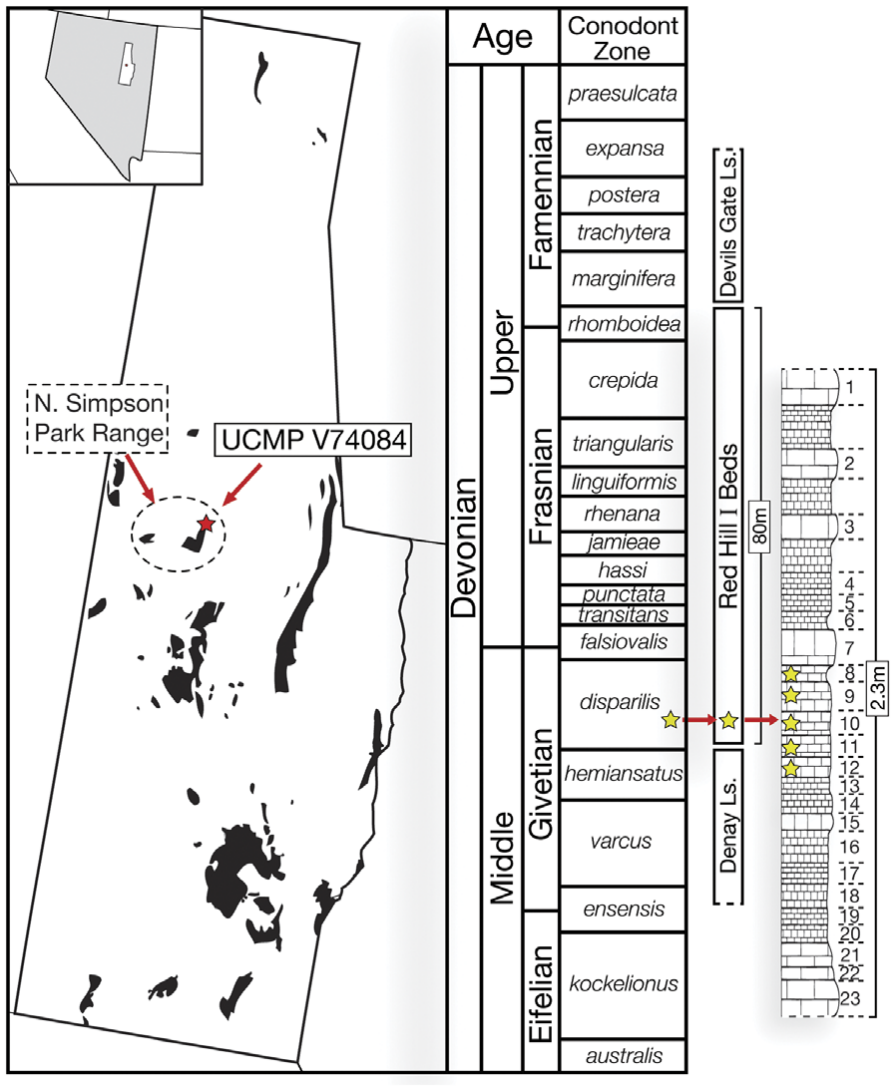
Geographic location and stratigraphic position of the Red Hill I field site (UCMP V74084) in Eureka Co., Nevada, USA. Black patterning within Eureka County represents exposed Devonian outcrops. Stars represent where the fossil material was collected. Red Hill I section courtesy of H.-P. Schultze.

The material was discovered and excavated in the mid-late 1970s by University of California, Berkeley paleontologist Joseph T. Gregory and his graduate students at a field site in northeastern Nevada known as Red Hill I. The Red Hill I Beds are a series of silty limy mudstones alternating with thick-bedded limestones, bounded below and above by the Denay and Devils Gate Formations, respectively [16]. This University of California Museum of Paleontology field site (UCMP V74084) is located in the northern Simpson Park Mountains in Eureka County, Nevada. Conodont biostratigraphy places Red Hill I in the lower *Klapperina disparilis* zone [16], [17], the late Givetian stage of the Middle Devonian. The described sarcopterygian material was recovered from levels 8–12 of the roughly 1.5 m thick sequence of vertebrate-bearing beds immediately above the Denay Limestone (Figure 1).

The fauna and geology indicate that the sedimentary rocks comprising Red Hill I were deposited in a marine environment. Cnidarians such as conulariids, a clade known elsewhere only from marine strata [18], are preserved in levels 21-5 (Figure 1). Moreover, the widespread deposition of limestone and shale along the western margin of Laurentia suggests that the regional geology of the northern Simpson Park Range represents an open marine paleoenvironment [19], and in particular the outer continental shelf [16], [20]. Trace fossils preserved between levels one and two suggest a short-term nearshore paleoenvironment [20].

## Results

### Systematic Paleontology

Sarcopterygii [21]; Rhipidistia [22], [23]; Tetrapodomorpha [24]; Eotetrapodiformes [13]; *Tinirau clackae* gen. et sp. nov. urn:lsid:zoobank.org:pub:5DEE6139-42E1-4995-BAB2-5E0461AA57A0.

### Etymology

Tinirau (tea-knee-/r/áu) is a character of legend in Polynesian culture and traces to islands located at approximately the same latitude as Nevada during the Middle Devonian. According to the Rarotonga and Mangaia Islanders, Tinirau was a half-man, half-fish lord of the ocean creatures [25]. The specific name *clackae* honors the Cambridge palaeontologist and former advisor Jenny Clack, for her contributions to our understanding of the earliest digited sarcopterygians.

### Holotype

**Figure 2:**
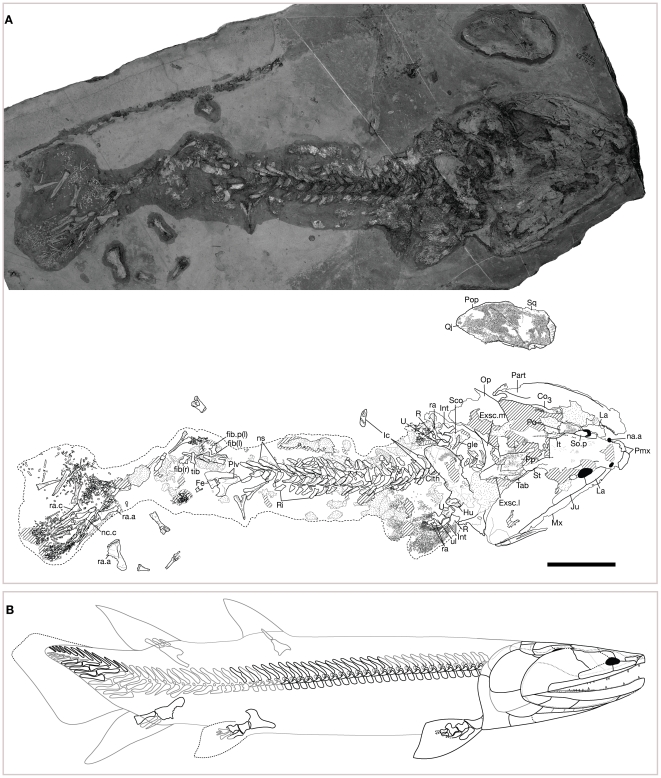
Holotype specimen UCMP 118605, interpretive drawing, and complete restoration of *Tinirau clackae*. (A) UCMP 118605, holotype, in dorsal, lateral and ventral view. See main text for details. Right is anterior. Scale bar equals 10 cm; (B) complete restoration; preserved elements outlined in black, inferred margins outlined in dashed black, hypothesized elements outlined in gray. See methods section for anatomical abbreviations. Note the reduced postaxial fibular processes on the fibulae (fib.p).

UCMP 118605, skull and postcranium (Figures 2, S1).

### Material

This description is based on six specimens (UCMP 117884, 118283, 118605, 123135, 190998, 190999) from a single locality. All specimens preserve complete or partial skull remains. Two specimens (UCMP 118605, 190999) preserve postcrania and appendicular elements in some degree of articulation. Specimens UCMP 118283 and 123135 were preserved in association with one another, adjacent on the same small block but not articulated. Not all specimens of *Tinirau* preserve every available character state, but consistent features among all specimens indicate that they represent a single taxon. These features include: elongate glenoid fossae (UCMP 118065, 190999), reduced posterior processes on the maxillae (UCMP 118065, 190999), fused parietals (UCMP 117884, 118238, 118065, 190999), fused anterior tectals and lateral rostrals (UCMP 11784, 118283), a row of non-fang teeth on the elongate posterior coronoids (UCMP 118605, 123135), and similar proportions and dentitions of the dermopalatines and entopterygoids (UCMP 190998, 190999).

### Locality

USA, Eureka Co., Nevada, Simpson Park Mountains north of the Denay Valley, UCMP locality V74084.

### Horizon

Lower *disparilis* conodont zone of the Red Hill I beds, immediately above the Denay Formation.

### Age

Middle Devonian, upper Givetian stage.

### Diagnosis

**Figure 3:**
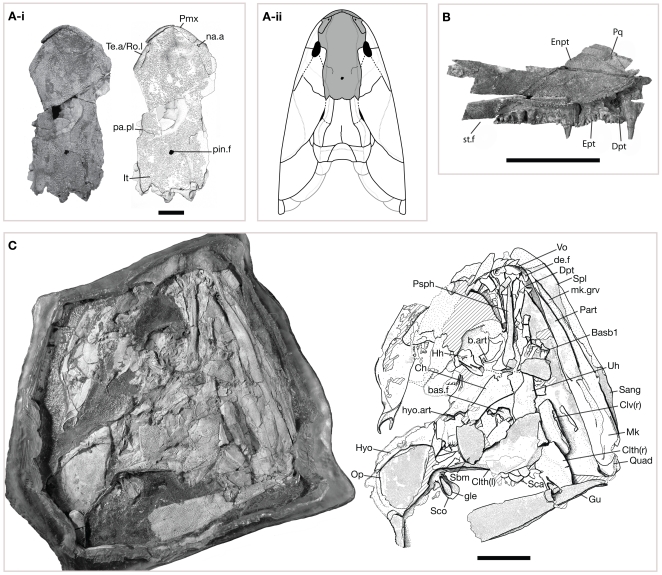
Ethmoid skull region and palate of *Tinirau clackae*. (A-i) UCMP 117884, ethmoid skull. Anterior is toward the top of the page. Scale bar equals 2 cm; (A-ii) dorsal skull reconstruction with infilled gray ethmoid region following from (A-i); (B) Left palatal fragment of UCMP 190998. Right is anterior. Scale bar equals 5 cm; (C) Skull, partial shoulder, and interpretive drawing of UCMP 190999. Uniform stipple covering distal jaw elements indicate unexposed portions of the specimen still covered by bioplastic; similarly, the dotted line posterior to the parasphenoid (Psph) notes the division between ethmoid and oticoccipital regions recovered from X-ray imaging. Anterior is toward the top of the page. Scale bar equals 5 cm. See methods section for anatomical abbreviations. Note the elongate glenoid fossa (gle) on the left scapulocoracoid (Sco).

An eotetrapodiform sarcopterygian distinguished from known tristichopterids by (*i*) an elongate posterior jugal process (Figures 2, S1), (*ii*) a dermal cheek plate with fused squamosal, preopercular, and quadratojugal elements (Figures 2, S1), (*iii*) deep tongue-and-groove embayments along the posteromedial margins of the intertemporals (Figures 3A, S2), (i*v*) fused anterior tectals with lateral rostrals (Figures 3A, S3), (*v*) medially straight anterior parietal margins in the unfused skull-table (Figure S3), (*vi*) a fused ethmoid skull-table in larger specimens—i.e., later ontogenetic stages (Figures 2, 3A, S1, S4), (*vii*) ectopterygoids that contribute to the subtemporal fossae (Figure 3B), (*viii*) splenials that remain unsutured to the prearticular (Figure 3C), and (*ix*) reduced postaxial fibular processes (Figures 2, S1, S6). Moreover, it is differentiated from elpistostegalians by (*I*) facially positioned anterior nostrils (Figure 3A), (*II*) a (inferred) lateral component to the ventral orbital margins (Figure 2, S1), (*III*) the presence of a median postrostral (Figure S3), (*IV*) the absence of frontal bones (Figures 2–3, S1, S3, S4), (*V*) the presence of a (anteriorly positioned) postspiracular (Figure S4), (*VI*) long posterior vomerine processes (Figure S2), (*VII*) an absence of jugal-quadratojugal contact (Figures 2, S1, 2), (*VIII*) a small scapulocoracoid (Figures 3C, S7), and (*IX*) round body scales (Figure 3C).

### Remarks

Tetrapodomorpha here defines total-group tetrapods, and I restrict the use of the term tetrapod to the crown-group. I use the monophyletic definition of Elpistostegalia [3], [26] to refer to the clade consisting of *Panderichthys* and crownward taxa. Moreover, following from the phylogenetic result presented below, I use Canowindridae as a stem-based name to refer to the clade constituting *Marsdenichthys, Canowindra*, *Koharalepis*, and *Beelarongia*, use the stem-based Megalichthyiformes [13] to reference the formerly paraphyletic (here recovered monophyletic, see supplementary information) ‘osteolepidids’, and apply the stem-based Tristichopteridae to define any taxon more closely related to *Tristichopterus* than to *Elpistostege*. In turn, I use ‘osteolepiform’ to encapsulate the grade of tetrapodomorph that includes canowindrids+megalichthyiforms+tristichopterids, and Eotetrapodiformes [13] as a node-based definition to refer to tristichopterids and elpistostegalians. Because of the curious morphology and phylogenetic position of the newly described taxon, I avoid calling this animal an elpistostegalian, and let future studies confirm or refute the phylogenetic hypothesis presented here. In addition, following from the revised phylogenetic placement of *Platycephalichthys bischoffi* [13], I refer to this taxon by its name only, as opposed to calling it a tristichopterid or an elpistostegalian.

### Comparative Description

The snout of *Tinirau* has one pair of facially positioned external nostrils as in all tetrapodomorphs except *Kenichthys* and elpistostegalians. However, in *Tinirau*, the nares penetrate a single, fused element consisting of the anterior tectal and lateral rostral (Figure 3A). Similar to ‘osteolepiforms’, *Platycephalichthys*, and elpistostegalians less crownward than *Ventastega*, the premaxilla forms a broad part of the choanal margin (Figure S2). Moreover, and differing from *Ventastega* and *Acanthostega*, a single median postrostral and several nasal bones create a solid snout lacking a dorsal fontanelle (Figures 2A, 3A, S1, S3).

The anterior skull roof of *Tinirau* is plesiomorphic among tetrapodomorphs: about 25% of the skull extends anterior to the mid-orbital margins (Figures 2A, 3A, S1). Such proportions are more similar to those of rhizodonts and canowindrids than to those of other eotetrapodiforms. The anterior-most paired roofing bones are the parietals, which are pierced by a pineal foramen that lies posterior to the orbits in larger specimens, or later ontogenetic stages (Figures 3A, S3). This condition is similar to early diverging ‘osteolepiforms’ such as *Koharalepis*, *Canowindra*, and *Gyroptychius*, and later-diverging tristichopterids more phylogenetically distal than *Eusthenopteron*. A functional dermal intracranial joint is unknown considering the tongue-and-groove articulations of the intertemporal and supratemporal bones that span this region. However, because the skull tends to be preserved in two parts, with the symplesiomorphic condition at least across the parietal/postparietal region, such a ‘joint’ is scored as present in *Tinirau* (Figures 2A, 3A, S1, S4). The condition in *Tinirau* is thus either autapomorphic (considering that dermal suturing in *Panderichthys* involves only the parietals and postparietals) or ‘intermediate’ because of the simultaneous suturing and simple abutment found across its dermal intracranial division. Interestingly, *Platycephalichthys* also has posteriorly recessed intertemporals suggesting a similar intracranial configuration [27].

The postparietal shield is not extremely wide posteriorly, as in canowindrids, nor do the parietals narrow to a point caudally, as in rhizodonts. Instead, the tabulars extend to the posterior margin of a postparietal shield that is approximately as wide as the ethmoid, a condition akin to that seen in tristichopterids, *Panderichthys*, *Tiktaalik*, and *Ventastega* (Figure S4). Lateral to the tabular resides a postspiracular ( = extratemporal) situated in the plesiomorphic anterior position, similar to the condition in Devonian tetrapodomorphs except tristichopterids phylogenetically distal of *Spodichthys* (Figure S4). The postspiracular is lost in known elpistostegalians.

Surrounding the orbit, the anterior and posterior supraorbitals ( = prefrontals and postfrontals) are of similar size and contact one another anterior to the mid-orbital margin. The posterior supraortbitals do not extend anterior to the orbits, similar to the condition in other Devonian tetrapodomorphs except a few late-diverging tristichopterids (Figures 2A, S1, S3). The lacrimal and jugal meet approximately at the mid-ventral orbital margin where, unlike in *Mandageria* and *Eusthenodon*, the postfrontal and lacrimal do not make contact (Figures 2A, S1). Moreover, unlike in elpistostegalians, the squamosal (here, bound up in a fused cheek plate) precludes abutting of the jugal and quadratojugal (Figures 2, S1, S4). It is not known *directly* if the postorbital contributes to the orbit of *Tinirau*, but based on the topology of this element and neighboring bones in UCMP 118605, it is inferred to make a minor contribution (Figures 2A, S1).

The jaws of *Tinirau* are characteristically eotetrapodiform in form, although contain a unique combination of plesiomorphic and apomorphic traits. The premaxillary teeth are all of similar size as in early diverging tristichopterids and elpistostegalians (Figure S2). However, the maxilla lacks a posterodorsal process, a state shared with *Platycephalichthys* and elpistostegalians such as *Panderichthys* on crownward, but also with derived tristichopterids such as *Cabonnichthys* and *Mandageria* (Figures 2A, S1, S4). Dentary fangs are present, similar to *Platycephalichthys* and elpistostegalians, though this character is also known in rhizodonts, megalichthyids, and derived tristichopterids (Figure 3C). The posterior coronoid is much longer than the anterior two coronoids, yet only carries one fang pair followed by a row or 5+ medium-sized teeth (Figure S5). This state combination is not present in any tristichopterid, and only shared with *Platycephalichthys* and early elpistostegalians such as *Panderichthys*. In other words, tristichopterids with long posterior coronoids also bear two posterior fang pairs, and those tristichopterids with one fang pair do not have very long posterior coronoids. A distinct Meckelian groove is visible in the lower jaw of UCMP 190999, and similar to the condition in non-elpistostegalian tetrapodomorphs, it bears an ossified posterior Meckelian region separating the prearticular/angular contact (Figure 3C).

The operculogular elements in UCMP 190999 are similar in shape and proportion to those of other Devonian ‘osteolepiforms’, and therefore are not diagnostic of a physical neck (i.e., a discrete, disconnecting region) between the shoulders and head (Figures 2A, S1, S4). Similar to *Kenichthys* and *Platycephalichthys*, a large preoperculum is sutured to the squamosal in a cheek plate and is also visible in visceral view in UCMP 118605 and 190999 (Figures 2A, S1, S4). The spiracular notch is not well-preserved, but judging from the narrow space between the squamosal and postparietal shield, it is inferred to be small and thus more like the condition in most ‘osteolepiforms’ rather than to that of *Gogonasus* and elpistostegalians (Figures 2A, 3A-ii, S1). The presence and size of a median gular remain unknown.

The palate of *Tinirau* is broadly similar to the tristichopterid condition, although it differs in a few interesting ways. As in tristichopterids, the posterior vomerine processes are long and underlap the parasphenoid substantially, although the latter condition is also present in *Panderichthys* and *Tiktaalik* (Figures 3C, S2). However, unlike in tristichopterids, the ectopterygoids contribute to the subtemporal fossae (Figure 3B). Among Devonian tetrapodomorphs, only the megalichthyiforms *Gogonasus* and *Medoevia*, and taxa crownward of tristichopterids, are known to have ectopterygoids that make this contribution. Moreover, and unlike all tristichopterids except *Spodichthys*, *Tinirau* retains the ancestral tetrapodomorph condition of bearing one ectopterygoid fang pair (Figure 3B). Such a condition is also retained in *Panderichthys* and *Tiktaalik*. As in tristichopterids and Devonian elpistostegalians, the anterior end of a densely denticulated entopterygoid resides considerably anterior to the processus ascendens of the palatoquadrate. This process is not preserved directly in *Tinirau*, but judging from the relative proportions of the palatoquadrate complex and of the positions of its associated articulations, this inference can be drawn with comfortable precision (Figures 3B–C, S2).

The neurocranium is plesiomorphic in many ways, although it shares some similarities with those of tristichopterids. A fully ossified ethmoid extends below a narrow tectum orbitale and articulates with its posterior otic-occipital counterpart via an endoskeletal intracranial joint. In turn, a basicranial fanestra spans this division (Figures 3C). These states are present in all Devonian tetrapodomorphs except for *Kenichthys* and taxa crownward of *Tiktaalik*. By contrast, *Tinirau* shares with tristichopterids a relatively anterior ventral hyomandibular facet (Figure 3C). In other words, this state is generally considered to diagnose tristichopterids, but is here reconstructed to be either convergent among these taxa, or to ancestrally diagnose eotetrapodiforms only primitively.

The cephalic branches of the sensory canal system are typical of most other Devonian tetrapdomorphs, although *Tinirau* retains a few traits—such as the postorbital junction of supra- and infraorbital canals, a line of continuous pores that comprise the mandibular canal, and a surangular pitline—that are otherwise lost in taxa crownward of *Tiktaalik* and *Acanthostega* (Figures 2A, S1, S5). As in *Glyptopomus*, *Marsdenichthys*, tristichopterids, *Platycephalichthys*, *Panderichthys*, and *Tiktaalik*, the sensory canals course through a tuberculate dermal skeleton that lacks the starburst ornamentation characteristic of the first digit-bearing elpistostegalians (Figures 3A-i, 3C, S3, S4, S5). Such elements also lack the thick ‘shine’ characteristic of cosmine-covered sarcopterygians such as megalichthyiforms.

The shoulder is typically tetrapodomorph in form, but it bears a few differences from those of key taxa. The anterior median extrascapular margin is “long” and therefore unlike those of canowindrids and *Mandageria* (Figures 2A, 3A-ii, S1, S4). A postbranchial lamina is present on the cleithrum (Figures 2A, S1), although posttemporals, supracleithra, anocleithra, and an interclavicle are not preserved. Unlike in elpistostegalians such as *Panderichthys* and *Tiktaalik*, a small scapulocorocoid is elevated from the ventral plane formed by the clavicles. However, the glenoid is relatively elongate and bears a medial ‘accessory’ region that is less reflexed than the condition seen in megalichthyiforms such as *Medoevia* and tristichopterids such as *Eusthenopteron* (Figures 2A, 3C, S1). Although the humerus is crushed, judging from the shape of the glenoids, it appears that the convex caput humeri retains less of the oblate shape than is typical of ‘osteolepiforms’. Such an elongate condition is more characteristic of elpistostegalians.

Paired appendages are only preserved in UCMP 118605 (Figures 2A, S1). The left humerus is crushed and situated below the cleithrum, but it articulates with the rest of a well-preserved pectoral limb. The right humerus is missing, but the elongate glenoid and distal pectoral elements remain. The pectoral limb is symplesiomorphic, and generally similar to the ‘osteolepiform’ condition. As in ‘osteolepiforms’ and elpistostegalians such as *Panderichthys* and *Tiktaalik*, the ulna is about half as long as the radius and articulates with an ulnare and intermedium. As in ‘osteolepiforms’, the ulnare retains a postaxial process and only articulates with two additional distal radials. Proximal lepidotrichia are about three times longer than more distal ones (Figures 2A, S1).

Caudally, the pelvis articulates with a femur that is preserved in association with the acetabulum, despite the disassociation of distal elements (Figures 2A, S1, S6). As in *Eusthenopteron*, the right and left disarticulated fibulae bear preaxial radial facets positioned about one half-step proximal to their postaxial counterparts. However, and unlike in *Gooloogongia* and *Eusthenopteron*, the postaxial fibular process is highly reduced and not simply the ‘fibula-equivalent’ of the condition seen in the ulnare. Interestingly, the pelvic limb of *Panderichthys* also displays a similar ‘lip’ overhanging the postaxial edge of the fibulare ([28], figure 1, pg. 1146).

The vertebral elements are preserved in near complete articulation, and are known only from UCMP 118605 (Figures 2A, S1). Paired intercentra are visible entirely in part/counterpart, and stout non-imbricate ribs radiate laterally, immediately posterior to the cleithrum. The axial skeleton proceeds through a left twist at ∼90° around mid-body, and posterior to the pelvis folds over itself so that the distal tip of the heterocercal caudal fin skeleton comes to face the more anterior (dorsal) neural spines. Paired pleurocentra are not preserved and are presumed to have been cartilaginous. There is no evidence for dorsal fin radials, although dorsal fins are hypothesized to have been present. By contrast, a dissociated anal fin basal and radial are preserved immediately dorsal to the caudal fin. The notochordal canal is visible and arches dorsally through the neural and haemal arches of the caudal fin skeleton (Figures 2A, S1).

## Discussion

### Phylogeny, Stratigraphy, and Evolutionary Patterns

**Figure 4:**
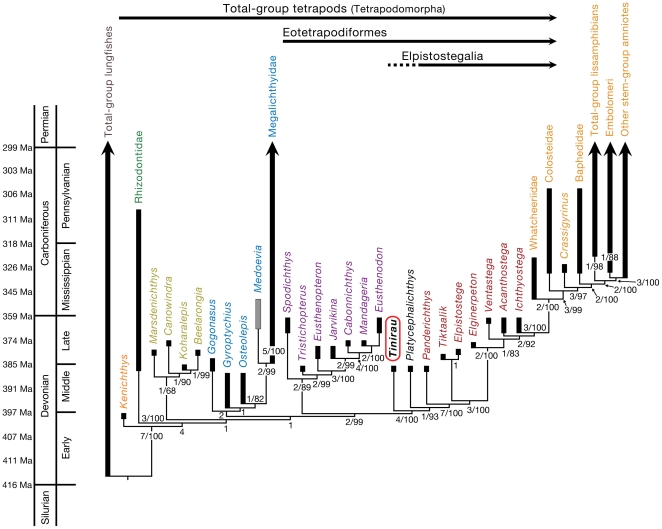
Interrelationships among Devonian and select Carboniferous tetrapodomorphs including new data from *Tinirau clackae*. Analysis includes 46 taxa and 204 characters. Tree length = 454, consistency index = 0.5572, retention index = 0.8481; consistency index excluding the four autapomorphic (uninformative) characters = 0.5532, retention index = 0.8481. Numbers corresponding to respective nodes represent: Bremer decay value/Bayesian posterior probability. Ghost ranges are calibrated after the early Middle Devonian (Eifelian) Zachełmie tracks ([15] and “scenario 1” from Friedman and Brazeau (2011). Tetrapodomorphs include all taxa that are not total-group lungfishes. Rhizodonts are in green, canowindrids are in yellow, megalichthyiforms are in blue, tristichopterids are in purple, Devonian elpistostegalians are in red, and Carboniferous elpistostegalians are in orange. The character list and data matrix are available as supplementary information.

A phylogenetic analysis using PAUP [29] recovered a single most parsimonious tree. A Bayesian analysis [30], [31] of the same data provided an additional metric. There are no major polytomies among the ‘osteolepiform’ grade taxa. Instead, the major clades, Rhizodontidae, Canowindridae, Megalichthyiformes, and Tristichopteridae form successive sister taxa to more crownward groups. *Tinirau* emerges as the sister to *Platycephalichthys* and elpistostegalians, one step crownward of tristichopterids (Figure 4).

The synapomorphies of *Tinirau* and crownward taxa include a pair of dentary fangs (Figure 3C), a posterior coronoid that is much longer than the anterior coronoids (Figures 2, S1, S5), an organized tooth row on the posterior coronoid (Figures 2, S1, S5), a weak posterodorsal maxillary process (Figures 2, S1, S4), a pineal foramen that lies posterior to the orbits (Figures 2–3A, S1), an elongate glenoid fossa (height∶width ratio 40–50%) (Figures 2–3C, S1, S7), and a reduced postaxial fibular process (Figures 2, S1, S6). Unsurprisingly, considering the widespread homoplasy among rhipidistians, nearly all of these characters evolved independently in at least one other group of Devonian tetrapodomorph, especially derived tristichopterids. Interestingly, previous studies that included *Platycephalichthys* recovered a similar pattern: synapomorphies that link *Platycephalichthys* and elpistostegalians also evolved in derived tristichopterids [13]. However, despite such parallelism, the phylogenetic result (Figure 4) is supported because of *Tinirau*'s unique combination of aforementioned apomorphies with an interesting array of symplesiomorphies—e.g., a single fang pair on the posterior coronoid (Figures 2, S1, S5), an anteriorly positioned postspiracular (Figure S4), a single ectopterygoid fang pair (Figure 2B), about 25% of the dermatocranium anterior to the orbits (comparison of specimens in Figures 2–3, S1), and a heterocercal caudal fin skeleton (Figures 2, S1) (see character optimizations in Text S1). Moreover, although the ectopterygoids of *Tinirau* contribute to the subtemporal fossae, the distribution of this trait in canowindrids and megalichthyiforms is too poorly known to be reconstructed as either symplesiomorphic among elpistostegalians, or as synapomorphic of *Tinirau* plus crownward taxa. Among Devonian tetrapodomorphs, only the megalichthyiforms *Gogonasus* and *Medoevia*, and eotetrapodiforms crownward of tristichopterids, have ectopterygoids that make this contribution.

This phylogenetic hypothesis implies that, (1) tristichopterid synapomorphies (see Text S1) have evolved in parallel during the early history of eotetrapodiforms; and (2) the 18+ elpistostegalian synapomorphies are cut in half (see Text S1) as taxa such as *Tinirau* and *Platycephalichthys* fill the graduated history of the tetrapod stem. This is predicted by current evidence, especially with the recent finding of marine, digit-bearing tracks that predate the earliest elpistostegalian body fossils by 10 Ma [15]. The discovery of *Tinirau* fills a phylogenetic gap missing from previous discoveries even though its stratigraphic range conforms with the timing of the body fossil record. Yet because ‘genus’-level preservation rates for Devonian tetrapodomorphs are an order of magnitude lower than ‘species’-level rates for groups considered to have dense records [32], the stratigraphic range of *Tinirau* is not surprising. Thus, when combined with the age of the trackways data, the late Middle Devonian (Givetian) age of *Tinirau*, its phylogenetic position as stem to the first digited forms, and its many symplesiomorphies may suggest a rich, yet undiscovered early tetrapodomorph record.

However, the phylogenetic distribution of potential sarcopterygian trackmakers does bring into question whether digited tetrapodomorphs even produced the Zachełmie trackways. Digit-bearing molds are preserved alongside continuous trackways but the ‘digits’ themselves are known only from isolated prints. Crown-group coelacanths, lungfishes, and tetrapods are known to engage in trotting gaits [33]–[35], and thus suggest substrate-based locomotor abilities in stem-tetrapods as well. Moreover, recent work [36] has shown that African lungfish using a bipedal pelvic-driven gait can produce the three trackways patterns known from the Zachełmie Quarry: (1) alternating doublets; (2) alternating singlets; and (3) opposite, ladder-like prints. Considering this range of potential explanations, the non-congruence of continuous digited prints with trackways patterns, and the increasingly strong stratophylogenetic congruence in the stem-tetrapod body fossil record, it might be wise to approach questions about timing of origins with a pluralistic eye and a bit of additional skepticism.

Questions about palaeoenvironment are more complicated, but *Tinirau*'s marine preservation is consistent with the marine influenced environments of the Zachełmie tracks and other closely related taxa [7], [37], [38], although likely not with others [3], [39].

### Evolutionary Morphology

Overall, the skeleton of *Tinirau* retains many ‘fish-like’ traits, but they are combined with a suite of elpistostegalian apomorphies. Because the utility of many of these characters remains obscure, here I elaborate on two traits that emerge as relevant to current discussions in tetrapodomorph evolution: the origins of the shoulder and pelvic limbs in the first digit-bearing elpistostegalians.

**Figure 5:**
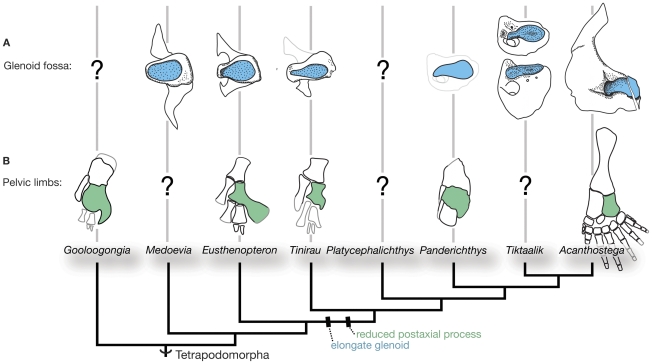
Glenoid fossae and pelvic limbs of select stem-tetrapods. Glenoids are illustrated in posterior view and highlighted in blue, fibulae are highlighted in green. The glenoid of *Tiktaalik* is depicted from two different perspectives, posterior view (above) and posteroventral view (below). The largest *Tiktaalik* specimens are more oblate, which may be autapomorphic relative to the condition in more crownward Devonian and Carboniferous taxa. The glenoid of *Panderichthys* was based on the shape of its caput humerus. See text for additional details. The in-plane glenoid measurement (height at maximum extent divided by maximum length) diagnoses an elongate glenoid fossa: *Medoevia* = 0.60; *Eusthenopteron* = 0.60; *Tinirau* = 0.42; *Panderichthys* = 0.48; *Tiktaalik* = 0.44; *Acanthostega* = 0.45.

As in tristichopterids such as *Eusthenopteron* and elpistostegalians such as *Panderichthys*, the shoulder of *Tinirau* retains the full osteichthyan complement of dermal and endochondral components. However, despite these general similarities, its glenoid is anteroposteriorly elongate and in this respect more similar to the condition found in *Panderichthys*, *Acanthostega*, and juvenile specimens of *Tiktaalik* (Figure 5A). Interestingly, glenoids in the largest *Tiktaalik* specimens are less elongate than those of smaller individuals [3], [40] (personal observations) and may reflect ontogenetic changes. Nonetheless, despite this possible autapomorphy, elongate glenoids in the first digited taxa correlate with parallel changes observed in the flattening of the caput humeri [2], [41]–[45]. Although the glenoids in *Medoevia*, *Eusthenopteron*, *Tinirau*, and *Panderichthys* have a strong posterior component, fossae in the former two taxa are more oblate than the condition present in the latter forms. This reinforces the hypothesis that mosaic changes in the pectoral limb began proximally before the distal portions acquired a more characteristic tetrapod-like morphology [46].

The femur, tibia, and fibula represent the only pelvic elements preserved in *Tinirau*, but they share an interesting similarity with *Panderichthys*, the only non-digit bearing elpistostegalian from which reasonable pelvic material is known [28]. One major difference between the fibulae of a rhizodont (e.g., *Gooloogongia*) or a tristichopterid (e.g., *Eusthenopteron*) and an elpistostegalian (e.g., *Panderichthys*) is that the postaxial process in *Panderichthys* is reduced to a mere lip or overhang bordering the posterior margin of the distal fibulare [4], [28], [47] (Figures 4B, S6). In this respect, the lack of a prominent postaxial process in the fibula of *Tinirau* is more similar to the condition observed in crownward taxa. This pattern underscores previous phylogenetic reconstructions of the appendicular skeleton in which conventional crown group limb characteristics first originate in the pelvic fins [48].

**Figure 6:**
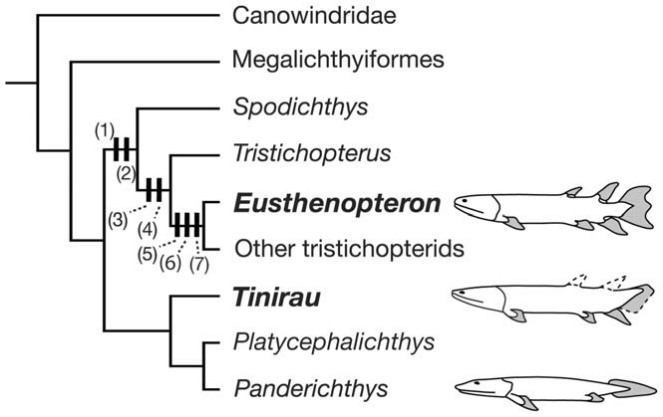
The nested phylogenetic position of *Eusthenopteron* within tristichopterids (*Spodichthys*+closest relatives) relative to *Tinirau*, *Platycephalichthys*, *Panderichthys*, and more crownward taxa. Character states supporting this topology: (1) a parasymphyseal plate not sutured to the anterior coronoid; (2) posterior coronoids longer than more anterior coronoids; (3) 33–40% of the dermatocranium anterior to the orbits; (4) a posteriorly displaced postspiracular; (5) posterior coronoids one third longer than the anterior coronoids; (6) two ectopterygoid fang pairs; and (7) a diphycercal caudal fin.

The new phylogeny also helps to displace *Eusthenopteron* as our iconic surrogate piscine ‘ancestor’. *Eusthenopteron* shares with other tristichopterids a sequence of traits that nest it well within tristichopterids and not immediately along the tetrapod stem (Figure 6). Instead, this result builds upon the work of Coates and Friedman (2010), whereby *Tinirau* and *Platycephalichthys* fill this position and provide an anatomical record on the transition to land. These taxa spread primitive tetrapodomorph traits along the early history of elpistostegalians, raise additional questions about when digited sarcopterygians first evolved, and fill a gap between tristichopterids and the first digited sarcopterygians in interesting and unexpected ways.

## Materials and Methods

### Phylogenetic Analysis

204 morphological characters were used to assess the phylogenetic position of the new taxon described above (*Tinirau clackae*) relative to other early tetrapodomorphs. Primary character sources [3], [8], [11], [13], [49]–[51] are indicated parenthetically following each character description in the supplementary information. Numbers following the citations refer to the character number in the original source. Characters modified from their original source are noted where applicable. Very few characters are shared between this analysis and Coates and Friedman (2010); this was intentional with the goal of assessing how largely independent data sets converge on a similar result.

Characters were polarized by comparison to outgroup taxa such as *Porolepis*, *Glyptolepis*, *Powichthys*, *Youngolepis*, *Diabolepis*, and *Dipterus*. These taxa were selected because they represent a range of total-group lungfish that are known from reasonable material, are well studied, and generally accepted as sister to total-group tetrapods.

Characters were coded based on a combination of published descriptions, specimen illustrations, and firsthand examination of fossil material. Care was taken to avoid simply recycling codings in the published literature. Specimens from the following museums were examined, and are noted following each taxon in the supplementary information: Australian Museum, Sydney (AMF), Australian National University (ANU), Geologisk Museum, Copenhagen, Denmark (MGUH), Latvian Museum of Natural History (LDM), Muséum national d'Histoire naturelle, Paris (MNHN), Museum Victoria, Melbourne, Australia (NMV), The Natural History Museum, London (MNH), Palaeontological Institute of the Russian Academy of Sciences, Moscow (PIN), National Museums of Scotland (NMS), Nunavut Fossil Vertebrate Collection (NUFV), Swedish Museum of Natural History, Stockholm (NR), University of California Museum of Paleontology (UCMP), University Museum of Zoology Cambridge (UMZC).

The data matrix was subjected to a maximum parsimony analysis in the software package PAUP 4.0b10 [29] and a Bayesian analysis using the software package Mr. Bayes 3.2 [30], [31]. All characters were assigned an equal weight, multistate characters were run unordered, and a heuristic search algorithm was used in PAUP to search for the shortest networks—rooted on *Porolepis*, *Glyptolepis*, *Powichthys*, *Youngolepis*, *Diabolepis*, and *Dipterus*. Bremer decay indices were calculated using PAUP [29] and TNT [52], [53], and Bayesian posterior probabilities were calculated with Mr. Bayes following an analysis that included 500,000 mcmc generations, sampling every 1,000 generations, and with 20 samples discarded as burnin. Character evolution was examined in MacClade [54], which was also used to produce the character state distributions in the supplementary information.

### Fossil Preparation

The material was prepared by an acid immersion procedure including baths of 30% formic acid or 10–20% acetic acid for 10–48 hours, followed by washing in running water for one month, and air-drying for 12–24 hours. Exposed elements were strengthened with glyptal or Duco cement. The three UCMP specimens 117884, 118283, and 123125 were studied 30 years ago by former UC Berkeley graduate student John Reed, although never published [55]. Because so much has changed in the record, systematics, and nomenclature of stem-tetrapods, it was necessary to redo the study completely.

### Nomenclatural Acts

The electronic version of this document does not represent a published work according to the International Code of Zoological Nomenclature (ICZN), and hence the nomenclatural acts contained in the electronic version are not available under that Code from the electronic edition. Therefore, a separate edition of this document was produced by a method that assures numerous identical and durable copies, and those copies were simultaneously obtainable for the purpose of providing a public and permanent scientific record, in accordance with Article 8.1 of the Code. The separate print-only edition is available on request from PLoS by sending a request to Public Library of Science, 1160 Battery Street, Suite 100, San Francisco, CA 94111, USA along with a check for $10 (to cover printing and postage) payable to ‘Public Library of Science’. In addition, this published work and the nomenclatural acts it contains have been registered in ZooBank, the proposed online registration system for the ICZN. The ZooBank LSIDs (Life Science Identifiers) can be resolved and the associated information viewed through any standard web browser by appending the LSID to the prefix ‘http://zoobank.org/’. The LSID for this publication is: urn:lsid:zoobank.org:pub:5DEE6139-42E1-4995-BAB2-5E0461AA57A0 *Tinirau clackae* Swartz gen. et sp. nov. For the genus, the LSID is: urn:lsid:zoobank.org:act:F459D126-AD40-4F69-A0C3-1B6885E891A7; and for the species, the LSID is: urn:lsid:zoobank.org:act:FBD69DA1-884C-4A2D-87C4-F7F8D85AB376.

### Anatomical Abbreviations

**ba.a**, anal basal; **b.art**, basal articulation of the basipterygoid process; **Basb1**, basibranchial #1; **bas.f**, basicranial fenestra; **Ch**, ceratohyal; **Clth**, cleithrum; **Clv**, clavicle; **Co_3_**, posterior coronoid; **de.f**, dentary fang; **Dpt**, dermopalatine; **Enpt**, entopterygoid; **Ept**, ectopterygoid; **Exsc.l**, lateral extrascapular; **Exsc.m**, median extrascapular; **Fe**, femur; **fib**, fibula; **fib.p**, posterior process of the fibula; **gle**, glenoid fossa; **Gu**, lateral gular; **Hh**, hypohyal; **Hu**, humerus; **Hyo**, hyomandibular; **hyo.art**, hyomandibular articulation; **Ic**, intercentrum; **Int**, intermedium; **It**, intertemporal; **Ju**, jugal; **La**, lacrimal; **Mk**, Meckelian bone; **mk.grv**, Meckelian groove; **Mx**, maxilla; **na.a**, anterior naris; **nc.c**, notochordal canal; **ns**, neural spine; **Op**, operculum; **pa.pl**, parietal pitline; **Part**, prearticular; **pin.f**, pineal foramen; **Plv**, pelvis; **Pmx**, premaxilla; **Po**, postorbital; **Pop**, preoperculum; **Pp**, postparietal; **Pq**, palatoquadrate; **Psph**, parasphenoid; **Qj**, quadratojugal; **Quad**, quadrate; **R**, radius; **ra**, radial; **ra.a**, anal radial; **ra.c**, caudal radial; **Ri**, rib; **Sang**, surangular; **Sbm**, submandibular; **Sca**, scale; **Sco**, scapulocoracoid; **Spl**, splenial; **St**, supratemporal; **st.f**, subtemporal fossa; **So.p**, posterior supraorbital; **Sq**, squamosal; **Tab**, tabular; **Te.a/Ro.l**, anterior tectal+lateral rostral; **tib**, tibia; **U**, ulna; **ul**, ulnare; **Uh**, urohyal; **Vo**, vomer. **(l)** or **(r)** refers to left or right when displaced from natural side.

## Supporting Information

Text S1 **Supplementary text.** Part A: taxa and characters used in the phylogenetic analysis; Part B: taxon-by-character matrix and character optimizations.
(PDF)

Figure S1 **Close-up of UCMP 118605 and specimen drawing.** UCMP 118605, holotype, in dorsal, lateral and ventral view. See main text for details; right is anterior. Abbreviations: **ba.a**, anal basal; **Clth**, cleithrum; **Clv**, clavicle; **Co_3_**, posterior coronoid; **Exsc.l**, lateral extrascapular; **Exsc.m**, median extrascapular; **Fe**, femur; **fib**, fibula; **fib.p**, posterior process of the fibula; **gle**, glenoid fossa; **Hu**, humerus; **Ic**, intercentrum; **Int**, intermedium; **It**, intertemporal; **Ju**, jugal; **La**, lacrimal; **Mx**, maxilla; **na.a**, anterior naris; **nc.c**, notochordal canal; **ns**, neural spine; **Part**, prearticular; **Plv**, pelvis; **Pmx**, premaxilla; **Po**, postorbital; **Pop**, preopercular; **Pp**, postparietal; **Qj**, quadratojugal; **R**, radius; **ra**, radial; **ra.a**, anal radial; **ra.c**, caudal radial; **Ri**, rib; **Sca**, scale; **Sco**, scapulocoracoid; **St**, supratemporal; **So.p**, posterior supraorbital; **Sq**, squamosal; **Tab**, tabular; **tib**, tibia; **U**, ulna; **ul**, ulnare; Scale bar equals 10 cm.
(TIF)

Figure S2 **Ethmoid palatal region and interpretive drawing of UCMP 117884.** Anterior is toward the top of the page. Abbreviations: **a.art**, autopalatine articulation; **b.art**, basal articulation of basipterygoid process; **cho**, choana; **‘cn’ II**, optic nerve; **It**, intertemporal, **nc**, neurocranium; **p.con**, processes connectens; **Pmx**, premaxilla; **pro.f**, profundus foramen; **Psph**, parasphenoid; **Vo**, vomer; **vo.f**, vomerine fang. ‘CN’ is in scare quotes because the optic nerve is not a real cranial nerve but a special-sensory extension of the diencephalon. Scale bar equals 5 cm.
(TIF)

Figure S3 **Ethmoid skull roof and interpretive drawing of juvenile specimen UCMP 118283.** Aside from the fusion of the anterior tectal and lateral rostral (similar to the adult specimen, UCMP 117884), many of the remaining roofing bones are unfused. The snout of this specimen is also proportionally shorter than the adult (when pineal foramina are aligned), suggesting substantial allometric change during ontogeny. In addition, it lacks the recessed tongue-and-groove articulations spanning the dermal intracranial joint, suggesting acquisition later in life. Anterior is toward the top of the page. Abbreviations: **It**, intertemporal; **Na**, nasal; **Pa**, parietal; **pin.f**, pineal foramen; **Pmx**, premaxilla; **Ro.p**, median postrostral; **So.a**, anterior supraorbital; **soc**, supraorbital canal; **Te.a/Ro.l**, (fused) anterior tectal/lateral rostral. Scale bar equals 5 mm.
(TIF)

Figure S4 **Skull, partial shoulder, and interpretive drawing of UCMP 190999.** Anterior is toward the top of the page. Abbreviations: **Clth**, cleithrum; **Clv**, clavicle; **De**, dentary; **Exsc.l**, lateral extrascapular; **Exsc.m**, median extrascapular; **Gu**, lateral gular; **Hyo**, hyomandibular; **Ju**, jugal; **La**, lacrimal; **Mx**, maxilla; **Op**, operculum; **Pa**, parietal; **Part**, prearticular; **Pop**, preoperculum; **Pp**, postparietal; **Psp**, postspiracular; **Qj**, quadratojugal; **Ro.p**, median postrostral; **Sco**, scapulocoracoid; **Sop**, suboperculum; **Sq**, squamosal; **St**, supratemporal; **Ta**, tabular; **Te.a/Ro.l**, (fused) anterior tectal/lateral rostral. (l) or (r) refers to left or right when displaced from natural side. Scale bar equals 5 cm.
(TIF)

Figure S5 **Lower Jaw of UCMP 123135.** (a) Dorsal view; (b) lateral view and interpretive drawing. Left is anterior. Abbreviations: **add.f**, adductor fossa; **Ang**, angular; **Art**, articular; **Co_1_**, anterior coronoid; **Co_2_**, middle coronoid; **Co_3_**, posterior coronoid; **co.f**, coronoid fang; **De**, dentary; **mc**, mandibular canal; **Pspl**, postsplenial; **Sang**, surangular; **Spl**, splenial. Scale bar equals 10 mm.
(TIF)

Figure S6 **Close-up of the pelvic region of UCMP 118605 highlighting the reduced postaxial fibular processes.** Abbreviations: **fe**, femur; **fib**, fibula; **Plv**, pelvis; **ns**, neural spine; **Ri**, rib. (l) or (r) refers to left or right. Scale bar equals 10 cm.
(TIF)

Figure S7 **Close-up of the elongate glenoid fossa of UCMP 190999.** Abbreviations: **Clth**, cleithrum; **gle**, glenoid fossa; **Sbm**, submandibular; **Sco**, scapulocoracoid. (l) refers to left. Scale bar equals 5 cm.
(TIF)
