# Supplementary material for: A Marine Stem-Tetrapod from the Devonian of Western North America
Source: PLoS One. 2012 Mar 20;7(3):e33683. doi: 10.1371/journal.pone.0033683 (PMC3308997; doi:10.1371/journal.pone.0033683)
Supplement: Text S1 — Supplementary text. Part A: taxa and characters used in the phylogenetic analysis; Part B: taxon-by-character matrix and character optimizations. (PDF) [file pone.0033683.s001.pdf]

Supplementary Information for:

A marine stem-tetrapod from the Devonian of western North America

Brian Swartz

Department of Integrative Biology and Museum of Paleontology

University of California, Berkeley, CA 94720, USA.

Brian.Darwin@Berkeley.edu

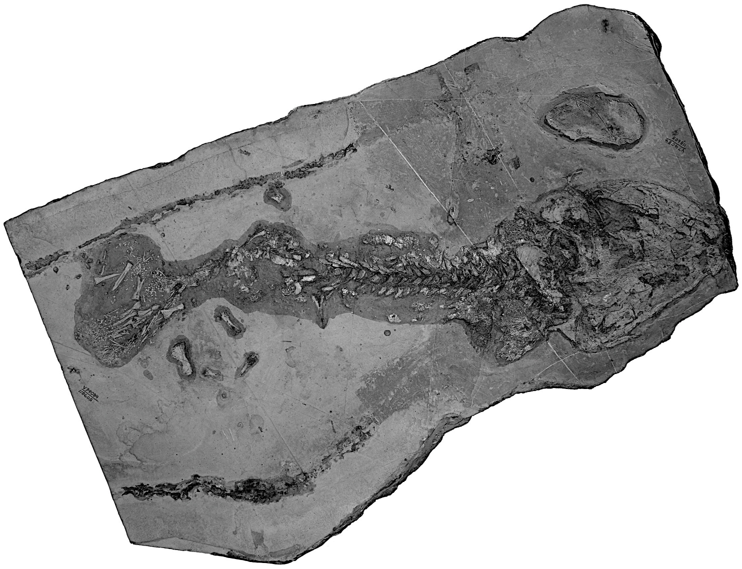

Supplementary Information

Supporting Text S1

Figures S1—S6

References

Supporting Text S1

Part A

Taxa and characters used in the phylogenetic analysis.—The following 204 morphological characters were used to assess the phylogenetic position of *Tinirau* relative to other early tetrapodomorphs. Not all specimens of *Tinirau* preserve every available character state. Consistent features among all specimens scored in this analysis that indicate they represent a single taxon, include: elongate glenoid fossa (UCMP 118065, 190999), reduced posterior process on the maxilla (UCMP 118065, 190999), fused parietals (UCMP 117884, 118238, 118065, 190999), fused anterior tectal and lateral rostral (UCMP 11784, 118283), a row of non-fang teeth on an elongate posterior coronoid (UCMP 118605, 123135), and similar proportions and dentition of the dermopalatines and entopterygoids (UCMP 190998, 190999). Total specimen lengths include: UCMP 118065 (97.8 cm), 190999 (26.5 cm), 117844 (15.4 cm), 190998 (12.1 cm), 123135 (4.7 cm), and 118283 (3.5 cm). These specimens probably represent 5 individuals. UCMP specimens 123135 and 118283 were preserved in association with one another, adjacent on the same small block but not articulated; they likely represent a single individual.

Primary character sources [1,2,3,4,5,6,7] are indicated parenthetically following each character description. Numbers following the citations refer to the character number in the original source. Characters modified from their original source are noted where applicable. Very few characters are shared between this analysis and Coates and Friedman (2010); this was maintained intentionally to demonstrate how nearly independent data sets converge on a similar result. This analysis also recovered a monophyletic Megalichthyiformes, with ‘osteolepidid’-grade tetrapodomorphs not simply emerging as lone lineages aligned as successive plesions to crownward forms. *Glyptopomus* was included in a separate analysis to test its influence on the phylogenetic result. Although its inclusion disrupted a monophyletic Canowindridae and pulled *Gyroptychius* and *Gogonasus* from Megalichthyiformes, it had no bearing on the phylogenetic position of any eotetrapodiform.

Characters were polarized by comparison to outgroup taxa such as *Porolepis*, *Glyptolepis*, *Powichthys*, *Youngolepis*, *Diabolepis*, and *Dipterus.* These taxa were selected because they represent a range of total-group lungfish that are known from reasonable material, are well studied, and generally accepted as sister to total-group tetrapods.

Characters were coded based on a combination of published descriptions, specimen illustrations, and firsthand examination of fossil material. Care was taken to avoid simply recycling codings in the published literature. Specimens from the following museums were examined, and are noted following each taxon below: Australian Museum, Sydney (AMF), Australian National University (ANU), Geologisk Museum, Copenhagen, Denmark (MGUH), Latvian Museum of Natural History (LDM), Muséum national d'Histoire naturelle, Paris (MNHN), Museum Victoria, Melbourne, Australia (NMV), The Natural History Museum, London (MNH), Palaeontological Institute of the Russian Academy of Sciences, Moscow (PIN), National Museums of Scotland (NMS), Nunavut Fossil Vertebrate Collection (NUFV), Swedish Museum of Natural History, Stockholm (NR), University of California Museum of Paleontology (UCMP), University Museum of Zoology Cambridge (UMZC).

Acanthostega [8,9,10,11,12,13,14] (MGUH f.n. 157, 255, 1227, 1258; UMZC T1291, T1300)
Balanerpeton [15] (UMZC T1312, T1313)
Baphetes [16,17,18,19]
Barameda [20,21,22] (NMV P10277, P160880, P160885, P212715)
Beelarongia [23] (NMV P160875, P160972)
Cabonnichthys [24] (AMF96856, F96858a, F96863, F96902, F98037, F98038)
Canowindra [25,26] (BMNH P.34420)
Cladarosymblema [27]
Crassigyrinus [28,29] (BMNH R10000; UMZC T1250)

Dendrerpeton [30,31,32,33] (UCMP 102367)
Diabolepis [34,35,36]
Dipterus [37,38] (BMNH P.17410, P.33165, P.34544, P.53507; MNHN GBP71, P72; NR P.3108, P.4827; UCMP 43714, 43727, 43728, 43729, 43730, 93066, 93067, 93068, 93069, 93070, 93071, 93072, 115246; UMZC GN1043)
Ectosteorhachis [39]
Elginerpeton [40,41,42]
Elpistostege [43] (BMNH P.60526 a,b)
Eoherepton [44,45]
Eusthenodon [46] (NR P.1475, P.1693)
Eusthenopteron [47,48] (BMNH P.60386, P.60388, P.60397; NR P.222, P.223, P249, P.287, P.290, P.330, P322 a,b, P.326b, P.382, P.2197, a,b, P2609, P.4611, P.6383; UMZC GN.790, GN.791, GN.797, GN.799)
Glyptolepis [49,50,51] (NR P.180, P.2503 a,b, P.8635)
Glyptopomus [52]
Gogonasus [7,53] (ANU 21885, 49259; NMV P221807)
Gooloogongia [54]
Greererepton [55,56,57,58] (UMZC T1220)
Gyroptychius [59,60,61] (MNHN GBP44, P63, P107, P138, P209, P264, P265, P307; NR P.1679, P.1698, P.4116, P.4220; UMZC GN.240, GN.939)
Ichthyostega [62,63] (MGUH 6055, 6064, 6081, MGUH f.n. 200, 300, 301)
Jarvikina [64]
Kenichthys [6,65]
Koharalepis [66]
Mandageria [67,68] (AMF96508, F96855a, F96857a,b,c, F98592c, F98593 a,b, F98594)
Marsdenichthys [69,70] (NMV P179619, P186572)
Medoevia [71]
Megalichthys [39,72,73,74,75,76] (NR P.6157; UMCZ GN.638)
Osteolepis [48,72,75,77] (MNHN GBP67, P186, P188, P195, P269 a,b, P277, P280, P284; NR P.1675, P. 4110, P.4139, P.11116; UCMP 43711, 43717, 43718, 43719, 43720, 43721, 43733, 58496, 58498, 58499)
Panderichthys [8,78,79,80,81,82,83,84] (NR P.6427; PIN 3547 [high resolution photograph]; LDM 60/123 [high resolution photograph])
Pederpes [85,86]
Platycephalichthys [4,64,87] (PIN 54/155, 54/156, 54/158, 54/159, 54/160, 54/160a, 54/161, 54/162, 54/163, 54/164, 54/165, 54/166, 54/183, 54/191, 54/192, 54/193, 54/194, 54/195 [high resolution photographs])
Porolepis [51,88] (MNHN SVD2001, 2034, 2158; NR A28633, A30483)
Powichthys [89,90,91]
Proterogyrinus [92,93]
Silvanerpeton [94] (UMZC T1317, T1351)
Spodichthys [61,95] (MGUH VP 6705 (P.1659), VP 6708 (P.1662), VP 6714 (P.1668), VP 6715 (P.1669))
Tiktaalik [5,96,97] (NUFV 108, 110)
Tristichopterus [98,99] (BMNH 66653, 66660, 66661, 66664, 66666, 66670; NMS.G.1875.29.220, G. 1875.29.221, G.1875.29.224, G.1875.29.225, G.1995.4.28; NR P.4196)
Ventastega [1,100]
Whatcheeria [101,102]
Youngolepis [103,104,105,106]

Characters

1. Ethmoid region

(Ahlberg et al. (2008): Character 25)

0 fully ossified
1 partly or wholly unossified

2. Rostral tubuli

(Coates and Friedman (2010): Character 1)

0 absent
1 present

3. Profundus foramen in postnasal wall

(Zhu and Ahlberg (2004): Character 81)

0 small
1 large

4. Fenestra ventrolateralis

(Coates and Friedman (2010): Character 5)

0 ventral to ethmoid articulation, in posterior view
1 extends dorsal to ethmoid articulation, in posterior view (post nasal wall unossified)

5. Pituitary vein exit

(Coates and Friedman (2010): Character 11)

0 anterior to basipterygoid process
1 dorsal to basipterygoid process

6. Extent of crista parotica

(Zhu and Ahlberg (2004): Character 33)

0 does not reach posterior margin of tabular
1 reaches posterior margin of tabular

7. Endoskeletal intracranial joint

(Coates and Friedman (2010): Character 14)

0 absent
1 present

8. Basicranial fenestra

(Zhu and Ahlberg (2004): Character 76)

0 absent
1 present

9. Processus descendens of sphenoid
(Zhu and Ahlberg (2004): Character 78)

0 absent
1 present

10. Posterior carotid opening in parasphenoid
(Zhu and Ahlberg (2004): Character 80)

0 large
1 small
2 absent

11. Tectum orbitale
(Zhu and Ahlberg (2004): Character 83)

0 narrow
1 extensive

12. Basipterygoid process
(Ahlberg et al. (2008): Character 24)

0 not strongly projecting with concave anterior face
1 strongly projecting with flat anterior face

13. Hypophysial region
(Ahlberg et al. (2008): Character 26)

0 solid side wall pierced by small foramina for pituitary vein and other vessels
1 single large foramen

14. Otic capsule lateral commissure bearing hyomandibular facets
(Ahlberg et al. (2008): Character 27)

0 present
1 absent

15. Relative positions of the hyomandibular facets
(Coates and Friedman (2010): Character 20)

0 dorsal directly above ventral
1 ventral anterior to dorsal
2 dorsal anterior to ventral

16. Parasymphysial plate
(Zhu and Ahlberg (2004): Character 1). *Platycephalichthys* scored after Snitting (2008b).

0 long, sutured to coronoid, denticulated or with tooth row
1 short, not sutured to coronid, denticulated
2 carrying tooth whorl

17. Parasymphysial plate dentition
(Modified from Ahlberg et al. (2008): Character 89)

0 Carrying a tooth whorl
1 shagreen or irregular tooth field
2 organised dentition aligned parallel to jaw margin

18. Parasymphsial fangs
(Modified from Ahlberg et al. (2008): Character 90)

0 absent
1 present

19. Parasymphysial plate: detachable whorl
(Zhu and Ahlberg (2004): Character 7)

0 detachable whorl
1 sutured plate with denticles or teeth

20. Lateral parasymphysial foramen
(Daeschler et al. (2006): Character 66)

0 absent
1 present

21. Mesial parasymphysial foramen
(Daeschler et al. (2006): Character 67)

0 absent
1 present

22. Length of dentary
(Zhu and Ahlberg (2004): Character 10)

0 long
1 short with lip fold

23. Dentary teeth
(Ahlberg et al. (2008): Character 85)

0 same size as maxillary teeth
1 larger than maxillary teeth
2 smaller than maxillary teeth

24. Accessory tooth rows on dentary

(Daeschler et al. (2006): Character 64)

0 present
1 absent

25. Dentary tooth row reaches symphysis
(Zhu and Ahlberg (2004): Character 11)

0 yes
1 no

26. Dentary fangs
(Modified from Zhu and Ahlberg (2004): Character 12)

0 absent
1 1 pair
2 1 unpaired (no replacement pit)

27. Dentary ventral edge
(Ahlberg et al. (2008): Character 55)

0 smooth continuous line
1 abruptly tapering or 'stepped' margin

28. Splenial
(Modified from Zhu and Ahlberg (2004): Character 2)

0 not sutured to prearticular
1 sutured to prearticular
2 postsplenial obstructing splenial-prearticular contact

29. Postsplenial suture with prearticular present
(Modified from Ahlberg et al. (2008): Character 69)

0 no
1 yes but interrupted by Meckelian foramina or fenestrae
2 uninterrupted suture

30. Postsplenial with mesial lamina
(Ahlberg et al. (2008): Character 67)

0 no
1 yes

31. Meckelian foramina/fenestrae, dorsal margins formed by
(Ahlberg et al. (2008): Character 63)

0 Meckelian bone
1 prearticular

2 infradentary

32. Meckelian foramina/fenestrae, height

(Ahlberg et al. (2008): Character 64)

0 much lower than adjacent prearticular
1 equal to or greater than depth of adjacent prearticular

33. Meckelian exposure in precoronoid fossa

(Daeschler et al. (2006): Character 65)

0 present
1 absent

34. Posterior coronoid longer than more anterior coronoids
0 no
1 yes

35. Posterior coronoid one-third longer than more anterior coronoids

(Modified from Zhu and Ahlberg (2004): Character 8)

0 no
1 yes

36. Coronoid fangs larger than marginal teeth

(Daeschler et al. (2006): Character 70)

0 yes
1 no

37. Coronoids: at least one carries shagreen

(Ahlberg et al. (2008): Character 80)

0 no
1 yes

38. Coronoids with a row of very small teeth or denticles lateral to tooth row

(Ahlberg et al. (2008): Character 81)

0 yes
1 no

39. Coronoids: size of teeth (excluding fangs) on anterior and middle coronoids relative to dentary tooth size

(Ahlberg et al. (2008): Character 82)

0 about the same
1 half height or less

40. Coronoid (anterior) contacts splenial
(Ahlberg et al. (2008): Character 49)

0 no
1 yes

41. Coronoid (middle) separated from splenial
(Ahlberg et al. (2008): Character 50)

0 yes, by prearticular
1 no
2 yes, by postsplenial

42. Coronoid (posterior) posterodorsal process
(Ahlberg et al. (2008): Character 52)

0 no
1 yes

43. Coronoid (posterior) posterodorsal process visible in lateral view
(Ahlberg et al. (2008): Character 53)

0 no
1 yes

44. Number of fang pairs on posteriormost coronoid
(Zhu and Ahlberg (2004): Character 13)

0 one
1 two
2 none

45. Non-fanged teeth on posterior coronoid
0 absent
1 organized tooth row
2 shagreen

46. Prearticular
(Zhu and Ahlberg (2004): Character 3)

0 not forked
1 forked

47. Prearticular sutures with mesial lamina of splenial
(Ahlberg et al. (2008): Character 71)

0 no, mesial lamina of splenial absent
1 yes
2 no, mesial lamina of splenial separated from prearticular by postsplenial

48. Prearticular-angular contact

(Ahlberg et al. (2008): Character 48)

0 separated by ventral exposure of Meckelian element
1 prearticular contacts angular edge to edge
2 mesial lamina of angular sutures with prearticular

49. Prearticular sutures with surangular

(Ahlberg et al. (2008): Character 70)

0 no
1 yes

50. Prearticular shagreen field, distribution

(Ahlberg et al. (2008): Character 92)

0 gradually decreasing from dorsal to ventral
1 well defined dorsal longitudinal band
2 scattered patches or absent

51. Prearticular with mesially projecting flange on dorsal edge along posterior border of adductor fossa

(Ahlberg et al. (2008): Character 73)

0 no
1 yes

52. Adductor crest

(Ahlberg et al. (2008): Character 47)

0 absent
1 peak anterior to adductor fossa, dorsal margin of fossa concave
2 peak above anterior part of adductor fossa, dorsal margin of fossa convex

53. Premaxillary tooth proportions

(Modified from Ahlberg et al. (2008): Character 38)

0 all approximately same size
1 enlarged anterior tooth
2 posteriormost teeth at least twice height of anteriormost teeth

54. Maxilla extends behind level of posterior margin of orbit

(Ahlberg et al. (2008): Character 12)

0 yes
1 no

55. Maxilla makes interdigitating suture with vomer

(Ahlberg et al. (2008): Character 10)

0 no
1 yes

56. Posterodorsal process of maxilla
(Zhu and Ahlberg (2004): Character 51)

0 present
1 very weak or absent

57. Vomer proportions
(Zhu and Ahlberg (2004): Character 22)

0 not much broader than long
1 much broader than long

58. Vomerine fangs
(Zhu and Ahlberg (2004): Character 24)

0 absent
1 present

59. Vomerine fang pairs noticeably smaller than other palatal fang pairs
(Ahlberg et al. (2008): Character 41)

0 no
1 yes

60. Vomerine row of small teeth
(Ahlberg et al. (2008): Character 43)

0 present
1 absent

61. Anterior wall of vomer (forming posterior margin of palatal fossa) bears tooth row meeting in midline
(Ahlberg et al. (2008): Character 42)

0 yes
1 no

62. Vomerine shagreen field
(Ahlberg et al. (2008): Character 44)

0 absent
1 present

63. Anteromedial process of vomer
(Zhu and Ahlberg (2004): Character 21)

0 absent, vomers separated
1 present
2 absent, vomers in close contact

64. Posterior process of vomers
(Zhu and Ahlberg (2004): Character 20)

0 absent
1 short
2 long

65. Relationship of vomer to parasphenoid
(Zhu and Ahlberg (2004): Character 23)

0 no contact (via small gap) or simple abutment
1 overlap
2 no contact via blockage by pterygoid elements

66. Parasphenoid, denticulated field
(Ahlberg et al. (2008): Character 29)

0 present
1 absent

67. Posterior end of parasphenoid
(Zhu and Ahlberg (2004): Character 26)

0 denticulated field extends into spiracular groove
1 denticulated field does not extend into spiracular groove

68. Parasphenoid
(Ahlberg et al. (2008): Character 28)

0 does not overlap basioccipital
1 overlaps basioccipital

69. Proportions of entopterygoid
(Zhu and Ahlberg (2004): Character 18)

0 anterior end level with processus ascendens
1 anterior end considerably anterior to processus ascendens

70. Entopterygoids meeting in midline
(Zhu and Ahlberg (2004): Character 19)

0 no
1 yes

71. Entopterygoid-quadrate ramus margin in the subtemporal fossa

(Ahlberg et al. (2008): Character 20)

0 concave
1 with some convex component

72. Dentition of palatoquadrate complex
(Zhu and Ahlberg (2004): Character 16)

0 marginal teeth
1 tooth plates

73. Entopterygoid shagreen
(Ahlberg et al. (2008): Character 37)

0 dense
1 a few discontinuous patches or absent

74. Anterior palatal fenestra
(Ahlberg et al. (2008): Character 93)

0 single
1 double
2 absent

75. Interentopterygoid vacuities
(Ahlberg et al. (2008): Character 95)

0 absent
1 at least 2 x longer than wide
2 < 2 x longer than wide

76. Dermopalatine exposure
(Modified from Ahlberg et al. (2008): Character 2)

0 more or less confined to margins of the tooth row
1 medial exposure in addition to the tooth row

77. Dermopalatine/ectopterygoid denticle row
(Ahlberg et al. (2008): Character 33)

0 present
1 absent

78. Dermopalatine/ectopterygoid shagreen field
(Ahlberg et al. (2008): Character 34)

0 absent
1 present

79. Ectopterygoid reaches subtemporal fossa
(Ahlberg et al. (2008): Character 4)

0 no
1 yes

80. Number of fangs on ectopterygoid
(Zhu and Ahlberg (2004): Character 17)

0 one pair
1 two pairs
2 none
3 one unpaired

81. Ectopterygoid row (3+) of smaller teeth
(Ahlberg et al. (2008): Character 32)

0 present
1 absent

82. Subterminal mouth
(Daeschler et al. (2006): Character 73)

0 absent
1 present

83. Number of nasals
(Zhu and Ahlberg (2004): Character 44)

0 many
1 one or two

84. Anterior tectal/septomaxilla
(Ahlberg et al. (2008): Character 1)

0 anterior tectal (external bone, dorsal to nostril)
1 septomaxilla (external or internal bone, posterior to nostril)
2 absent

85. Lateral rostral present
(Ahlberg et al. (2008): Character 9)

0 yes
1 no

86. Median postrostral
(Zhu and Ahlberg (2004): Character 43)

0 absent (postrostral mosaic)

1 present
2 absent (nasals meet in midline)

87. Dorsal fontanelle on snout
(Ahlberg et al. (2008): Character 94)

0 absent
1 present

88. Internasal pits
(Zhu and Ahlberg (2004): Character 25)

0 undifferentiated
1 strong midline ridge but shallow pits
2 deep pear-shaped pits

89. External nostrils
(Zhu and Ahlberg (2004): Character 29)

0 two pairs
1 one pair

90. Premaxilla forms part of choanal margin
(Ahlberg et al. (2008): Character 17)

0 broadly
1 point
2 not, excluded by vomer

91. Position of anterior external nostril
(Zhu and Ahlberg (2004): Character 30)

0 facial
1 edge of mouth

92. Lacrimal
(Ahlberg et al. (2008): Character 8)

0 contributes to orbital margin
1 excluded from margin

93. Contact between lacrimal and posterior supraorbital [postfrontal]
(Zhu and Ahlberg (2004): Character 56)

0 absent
1 present

94. Jugal
(Ahlberg et al. (2008): Character 7)

0 does not extend anterior to orbit
1 extends anterior to orbit

95. Jugal extends anterior to middle of orbit
(Daeschler et al. (2006): Character 78)

0 no
1 yes

96. Jugal-quadratojugal contact
(Zhu and Ahlberg (2004): Character 52)

0 absent
1 present

97. Position of orbits
(Zhu and Ahlberg (2004): Character 59)

0 lateral and widely separated
1 dorsal and close together

98. Postorbital bone
(Zhu and Ahlberg (2004): Character 54)

0 contributes to orbital margin
1 excluded from orbital margin

99. Contact between postorbital and lacrimal
(Daeschler et al. (2006): Character 84)

0 absent
1 present

100. Quadratojugal, squamosal and preopercular fused
(Zhu and Ahlberg (2004): Character 62)

0 no
1 yes

101. Subsquamosals
(Zhu and Ahlberg (2004): Character 61)

0 absent
1 present

102. Preoperculosubmandibular
(Zhu and Ahlberg (2004): Character 60)

0 absent
1 present

103. Width of ethmoid relative to its length, from snout tip to the posterior margin of the parietals
(Modified from Zhu and Ahlberg (2004): Character 32)
States based on clumped morphospace.

0 greater or = 80%
1 75%-45%
2 less than or = 35%

104. Proportion of skull roof (measured as length from tip of snout to posterior margin of postparietals) lying anterior to middle of orbits
(Modified from Daeschler et al. (2006): Character 75)
States based on clumped morphospace.

0 20-30%
1 33-40%
2 45-48%
3 >53%

105. B-bone
(Zhu and Ahlberg (2004): Character 46)

0 absent
1 present

106. Prefrontal (anterior supraorbital)
(Ahlberg et al. (2008): Character 15)

0 twice as long as broad, or less
1 three times as long as broad

107. Prefrontal (anterior supraorbital)
(Ahlberg et al. (2008): Character 16)

0 transverse anterior suture with tectal (or opens broadly into external nostril)
1 tapers to point anteriorly

108. Relative size of prefrontal [anterior supraorbital] and posterior supraorbital [postfrontal]
(Daeschler et al. (2006): Character 76)

0 similar
1 prefrontal much bigger

109. Postfrontals [posterior supraorbitals] extend anterior of orbits
(Daeschler et al. (2006): Character 86)

0 no

1 yes

110. Shape of postfrontals (posterior supraorbitals)
(Zhu and Ahlberg (2004): Character 58)

0 posterior process shorter than orbital margin
1 posterior process much longer than orbital margin

111. Contact between intertemporal and postfrontal (posterior supraorbital)
(Zhu and Ahlberg (2004): Character 55)

0 present
1 absent

112. Contact between parietal and postfrontal (posterior supraorbital)
(Zhu and Ahlberg (2004): Character 57)

0 present
1 absent

113. Frontals
(Zhu and Ahlberg (2004): Character 45)

0 absent
1 present

114. Parietals surround pineal foramen/eminence
(Zhu and Ahlberg (2004): Character 38)

0 yes
1 no

115. Pineal foramen
(Zhu and Ahlberg (2004): Character 36)

0 present
1 absent

116. Position of pineal foramen/eminence
(Zhu and Ahlberg (2004): Character 37)

0 level with posterior margin of orbits
1 well posterior to orbits

117. Shape of pineal series
(Zhu and Ahlberg (2004): Character 39)

0 round or oval
1 kite-shaped with distinct posterior corner. (non-applicable for *Kenichthys*)

118. Intemporal
(Ahlberg et al. (2008): Character 6)

0 present
1 absent

119. Dermal intracranial joint
(Zhu and Ahlberg (2004): Character 71)

0 present
1 absent

120. Postparietals narrow to a point posteriorly
(Zhu and Ahlberg (2004): Character 41)

0 no
1 yes

121. Proportions of postparietal shield
(Zhu and Ahlberg (2004): Character 40)

0 not extremely wide posteriorly
1 extremely wide posteriorly

122. Supratemporal
(Modified from Ahlberg and Johanson (1998): Character 49)

0 recognizable as a distinct bone
1 fused with postparietal

123. Posterior margin of tabulars
(Modified from Zhu and Ahlberg (2004): Character 42)

0 anterior to the posterior margin of postparietals
1 level with the posterior margin of postparietals
2 posterior to the posterior margin of the postparietals

124. Postspiracular (extratemporal)
(Zhu and Ahlberg (2004): Character 50)

0 present
1 absent

125. Position of the postspiracular (extratemporal)
0 anterior
1 posteriorly displaced

126. Contact between postspiracular [extratemporal] and supratemporal

(Zhu and Ahlberg (2004): Character 48)

0 absent
1 present

127. Premaxilla canal-bearing
(Zhu and Ahlberg (2004): Character 68)

0 yes
1 no

128. Infraorbital canal follows premaxillary suture
(Zhu and Ahlberg (2004): Character 69)

0 no
1 yes

129. Postotic sensory canal
(Zhu and Ahlberg (2004): Character 66)

0 runs through skull roof
1 follows edge of skull roof

130. Postorbital junction of supraorbital and infraorbital canals
(Zhu and Ahlberg (2004): Character 67)

0 absent
1 present

131. Mandibular sensory canal
(Ahlberg et al. (2008): Character 57)

0 prsesnt
1 absent

132. Mandibular canal exposure
(Ahlberg et al. (2008): Character 58)

0 entirely enclosed, opens through lines of pores
1 mostly enclosed, short sections of open grooves
2 mostly open, short sections with lines of pores
3 entirely open

133. Mandible: oral sulcus/surangular pit line
(Ahlberg et al. (2008): Character 59)

0 present
1 absent

134. Foramina (similar to infradentary foramina) on cheekplate
(Zhu and Ahlberg (2004): Character 63)

0 absent
1 present

135. Submandibulars and gulars
(Daeschler et al. (2006): Character 80)

0 present
1 absent

136. Large median gular
(Modified from Daeschler et al. (2006): Character 81)

0 absent
1 present

137. Preopercular
(Daeschler et al. (2006): Character 88)

0 large
1 small

138. Preopercular
(Ahlberg et al. (2008): Character 18)

0 present
1 absent

139. Opercular
(Ahlberg et al. (2008): Character 14)

0 present
1 absent

140. Spiracular notch
(Daeschler et al. (2006): Character 87)

0 absent
1 small opening
2 narrow groove
3 wide notch

141. Anterior margin of median extrascapular
(Zhu and Ahlberg (2004): Character 65)

0 long
1 very short

142. Extrascapular bones
(Zhu and Ahlberg (2004): Character 64)

0 median overlaps laterals
1 laterals overlap median

143. Extrascapular bones
(Daeschler et al. (2006): Character 77)

0 present
1 absent

144. Posttemporal
(Ahlberg et al. (2008): Character 109)

0 present
1 absent

145. Supracleithrum
0 present
1 absent

146. Anocleithrum
(Zhu and Ahlberg (2004): Character 85)

0 exposed
1 subdermal

147. Anocleithrum
(Ahlberg et al. (2008): Character 99)

0 oblong with distinct anterior overlap area
1 drop-shaped with no anterior overlap area
2 absent

148. Orientation of cleithrum
(Daeschler et al. (2006): Character 105)

0 vertically oriented: tilted less than 10 degrees caudally
1 angulated: tilted over 10 degrees caudally

149. Cleithrum, postbranchial lamina
(Ahlberg et al. (2008): Character 101)

0 present
1 absent

150. Contact margin for clavicle on cleithrum

(Zhu and Ahlberg (2004): Character 84)

0 straight or faintly convex
1 strongly concave

151. Scapulocoracoid
(Ahlberg et al. (2008): Character 115)

0 small and tripodal
1 large plate pierced by large coracoid foramen
2 very large plate without large coracoid foramen

152. Coracoid plate
(Daeschler et al. (2006): Character 103)

0 absent
1 present and extends ventromedially

153. Scapular blade
(Ahlberg et al. (2008): Character 114)

0 absent
1 small with narrow top
2 large with broad top

154. Shoulder joint polarity
(Zhu and Ahlberg (2004): Character 87)

0 caput humeri concave
1 caput humeri convex

155. Glenoid position
(Daeschler et al. (2006): Character 104)

0 elevated from plane formed by clavicles
1 offset ventrally to lie at same level as clavicular plane

156. Glenoid orientation
(Daeschler et al. (2006): Character 110)

0 posterior orientation
1 lateral component to glenoid orientation

157. Glenoid proportions
Measured in plane with glenoid orientation. Height at maximum extent divided by maximum length: *Medoevia* = 0.60; *Eusthenopteron* = 0.60; *Tinirau* = 0.42; *Panderichthys* = 0.48; *Tiktaalik* = scored as ?; *Acanthostega* = 0.45; *Ichthyostega* = 0.44; *Greererpeton* = 0.44; *Proterogyrinus* = 0.40; *Eoherpeton* = 0.47.

0 height/width ratio 60% or greater
1 height/width ratio 40-50%

158. Interclavicle
(Ahlberg et al. (2008): Character 106)

0 small and concealed (unornamented) or absent
1 large and exposed (ornamented)

159. Interclavicle shape
(Ahlberg et al. (2008): Character 107)

0 ovoid
1 kite-shaped
2 with posterior stalk

160. Archipterygial pectoral fin
(Zhu and Ahlberg (2004): Character 86)

0 no
1 yes

161. Humerus
(Ahlberg et al. (2008): Character 103)

0 narrow tapering entepicondyle
1 square or parallelogram-shaped entepicondyle

162. Body of humerus
(Zhu and Ahlberg (2004): Character 89)

0 cylindrical
1 flattened rectangular

163. Deltoid and supinator process on humerus
(Zhu and Ahlberg (2004): Character 90)

0 absent
1 present

164. Anterior termination of ventral ridge
(Daeschler et al. (2006): Character 96)

0 adjacent to the caput humeri
1 offset distally toward the proximodistal mid-region of anterior margin of humerus

165. Ectepicondylar process
(Daeschler et al. (2006): Character 100)

0 terminates proximal to epipodial facets
1 extends distal to epipodial facets

166. Radius and ulna
(Ahlberg et al. (2008): Character 110)

0 radius much longer than ulna
1 approximately equal length

167. Radial facet
(Daeschler et al. (2006): Character 98)

0 faces distally
1 has some ventrally directed component

168. Area proximal to radial facet
(Daeschler et al. (2006): Character 102)

0 short, cylindrical leading edge, with no muscle scars
1 enlarged, sharp leading edge, with areas for muscle attachments

169. Shape of radius
(Daeschler et al. (2006): Character 94)

0 bladelike
1 subcylindrical

170. Radial length
(Daeschler et al. (2006): Character 101)

0 longer than humerus
1 shorter than humerus

171. Ulnar facet
(Daeschler et al. (2006): Character 99)

0 faces distally
1 has some ventrally directed component

172. Olecranon process on ulna
(Daeschler et al. (2006): Character 95)

0 absent
1 present

173. Transverse joint at the level of the ulnare, intermedium and radius
(Daeschler et al. (2006): Character 89)

0 absent

1 present

174. Articulations for more than two radials on ulnare
(Daeschler et al. (2006): Character 90)

0 absent
1 present

175. Postaxial process on ulnare
(Daeschler et al. (2006): Character 91)

0 absent
1 present

176. Branched radials distal to the ulnare
(Daeschler et al. (2006): Character 93)

0 absent
1 present

177. Radials
(Zhu and Ahlberg (2004): Character 91)

0 jointed
1 unjointed

178. Digits
(Ahlberg et al. (2008): Character 102)

0 absent
1 present

179. Lepidotrichia in paired appendages
(Ahlberg et al. (2008): Character 108)

0 present
1 absent

180. Basal segments of lepidotrichia elongated
(Zhu and Ahlberg (2004): Character 92)

0 no
1 yes

181. Expanded ribs
(Daeschler et al. (2006): Character 113)

0 absent
1 present

182. Imbricate ribs
(Daeschler et al. (2006): Character 114)

0 absent
1 present

183. Ribs, trunk
(Ahlberg et al. (2008): Character 111)

0 no longer than diameter of intercentrum
1 longer

184. Ribs, trunk
(Modified from Ahlberg et al. (2008): Character 112)

0 all straight
1 at least some ventral component

185. Ribs, trunk
(Ahlberg et al. (2008): Character 113)

0 all cylindrical
1 some or all bear flanges from posterior margin which narrow distally
2 some or all flare distally

186. Supraneural spines
(Zhu and Ahlberg (2004): Character 99)

0 present
1 absent

187. Ilium, iliac canal
(Ahlberg et al. (2008): Character 104)

0 absent
1 present

188. Ilium, posterior process
(Ahlberg et al. (2008): Character 105)

0 oriented posterodorsally
1 oriented approximately horizontally posteriorly

189. Postaxial process on fibula
0 present
1 absent

190. Postaxial process on fibula, size

0 large
1 small

191. Dorsal and anal fins
(Zhu and Ahlberg (2004): Character 93)

0 present
1 absent

192. Posterior radials in posterior dorsal fin
(Zhu and Ahlberg (2004): Character 94)

0 not branched
1 branched

193. Caudal fin
(Zhu and Ahlberg (2004): Character 95)

0 heterocercal
1 diphycercal

194. Epichordal radials in caudal fin
(Zhu and Ahlberg (2004): Character 96)

0 absent
1 present

195. Nature of dermal ornament
(Ahlberg et al. (2008): Character 97)

0 tuberculate
1 fairly regular pit and ridge
2 irregular
3 absent or almost absent

196. Nature of ornament: "starbursts" of radiating ornament on at least some bones
(Ahlberg et al. (2008): Character 98)

0 no
1 yes

197. Cleithral ornamentation
(Daeschler et al. (2006): Character 106)

0 present
1 absent

198. Basal scutes
(Zhu and Ahlberg (2004): Character 97)

0 absent
1 present

199. Body scale morphology
(Zhu and Ahlberg (2004): Character 98). *Platycephalichthys* scored after Snitting (2008b).
0 rhomboid with internal ridge
1 round

200. Squamation
(Ahlberg et al. (2008): Character 117)
0 complete body covering of scales
1 ventral armour of gastralia

201. Tooth folding
(Zhu and Ahlberg (2004): Character 14)
0 none
1 generalized polyplocodont
2 labyrinthodont
3 dendrodont

202. Cosmine
(Zhu and Ahlberg (2004): Character 34)
0 present
1 absent

203. Westoll lines
(Zhu and Ahlberg (2004): Character 35)
0 absent
1 present

204. Hyomandibula orientation
(Long et al. (2006): Character 4)
0 posteroventral, distally terminating near jaw joint
1 almost horizontal orientation, opercular process high up dorsally
2 very short, laterally directed

Part B

Taxon-by-character matrix and character optimizations.—The data matrix was subjected to a maximum parsimony analysis in the software package PAUP [107] and a Bayesian analysis using the software package Mr. Bayes[108,109]. All characters were assigned an equal weight, multistate characters were run unordered, and a heuristic search algorithm was used in PAUP to search for the shortest networks—rooted on *Porolepis*, *Glyptolepis*, *Powichthys*, *Youngolepis*, *Diabolepis*, and *Dipterus*. Bremer decay indices were calculated using PAUP[107] and TNT[110,111], and Bayesian posterior probabilities were calculated with Mr. Bayes following an analysis that included 500,000 mcmc generations, sampling every 1,000 generations, and with 20 samples discarded as burnin. Character evolution was examined in MacClade[112], which was also used to produce the character state distributions below. *Eusthenopteron* is scored as *E. foordi* and *Platycephalichthys* scored as *P. bischoffi*.

A = 0 & 1; **B** = 0 & 2

	1		11		21		31		41	
Acanthostega	100-?	00002	0111?	02110	10010	10100	10100	10011	00-21	11001
Balanerpeton	10?-?	?0002	01???	-?1-0	00110	21221	20100	11-11	2??22	02202
Baphetes	10?-?	?0002	0111?	?111?	?0??0	0????	?????	1????	?????	?????
Barameda	00???	?1???	?????	?????	?0000	10???	?????	?????	?????	?????
Beelarongia	?0???	?????	?????	?????	?0???	?0???	?????	?????	?????	?????
Cabonnichthys	?0???	?1?02	0????	1????	?0000	101??	??0-1	00000	??-10	0???0
Canowindra	?0???	?????	?????	?????	?0???	?0???	?????	?????	?????	?????
Cladarosymblema	00000	01102	00000	01010	00000	10100	00000	00000	?0-00	00000
Crassigyrinus	?0???	?0002	0????	?2110	00110	?1100	10111	11-10	0???2	?1202
Dendrerpeton	10?-?	?0002	0111?	?????	?011?	?1?11	11???	?1-?1	?11??	??212
Diabolepis	01???	?0?0?	10?0?	?---?	?1?-?	0-?--	?????	-????	?????	?????
Dipterus	010??	?00?0	10???	?---?	?1?-?	0-0--	?????	-????	?????	??0??
Ectosteorhachis	00???	01102	?0??0	01010	00?00	10100	??000	00000	?0-00	00000
Elginerpeton	?????	?????	?????	?2111	10?00	10?00	001??	10000	0???1	11001
Elpistostege	?0???	?????	?????	?????	?????	?0???	?????	?????	?????	?????
Eoherepton	10?-?	?0002	01?1?	?????	??01?	?1111	10100	11011	0??22	01?12
Eusthenodon	?????	?????	0????	1??1?	?0?00	101??	???-1	0?00?	???10	0????
Eusthenopteron	00000	11102	00001	11010	00000	00100	000-1	00000	00-10	00000
Glyptolepis	00101	11101	00???	20-00	00001	00?00	??000	00000	???00	00000
Glyptopomus	?????	?1???	?????	?????	?00??	?????	?????	?????	?????	?????
Gogonasus	00000	01102	00000	01010	00000	00000	00000	000A0	???01	?0000
Gooloogongia	?0???	?1???	?????	01010	00000	1????	??0??	0???0	?????	?????
Greererepton	10?-?	?0002	0111?	?2110	00110	21100	111??	11111	01122	11202
Gyroptychius	?0???	01102	0??02	01010	00000	?0???	???00	00000	?0-00	?0?0?
Ichthyostega	10?-?	?0002	01?1?	02110	10210	10100	00100	10111	00-21	11102
Jarvikina	???0?	11102	00??1	1????	?0?00	001??	???-1	0????	?001?	0????
Kenichthys	000??	?1?02	10???	01010	00000	00000	??000	010A0	?0-00	000??
Koharalepis	?0???	?????	?????	?????	?000?	?0???	?????	?????	?????	?????
Mandageria	00???	11102	???02	?????	?0000	101??	?????	0?00?	?????	0????
Marsdenichthys	?????	?????	?????	?????	?0??0	00???	???00	0?0??	?????	?????
Medoevia	00000	01102	00000	01010	000?0	0?100	00000	00000	00-00	00000
Megalichthys	00???	?1102	00000	01010	00000	10100	??000	00000	?0-00	0000?
Osteolepis	00???	01102	00000	01010	00000	00?00	??0??	0????	?????	?????
Panderichthys	00010	01102	00000	01010	00000	10100	000-1	00000	00-01	00100
Pederpes	?0???	?0??2	01?1?	?????	?????	?????	?????	?????	?????	?????
Platycephalichthys	0??1?	?1???	?0???	01010	000?0	10???	000?1	00??0	00-01	000?0
Porolepis	?0101	11101	10?0?	20-00	00001	000??	???00	?0000	???00	00000
Powichthys	011?1	?1111	10?0?	20-0?	?0?01	0?0??	???00	??0??	???00	0?0??
Proterogyrinus	10?-?	?0002	01???	??110	00010	?1111	111??	11-11	???22	??212
Silvanerpeton	?0???	?0002	01???	?????	?0010	?1??1	101?0	11-1?	0??22	????2
Spodichthys	00???	11102	00001	1??1?	?0000	00100	00010	00??0	00-00	0000?
Tiktaalik	000-?	01102	00?00	0?010	00000	101?0	?00-1	00100	00-21	00100
Tinirau	0000?	?1102	00001	?????	?0000	10000	???-1	00000	00-01	00000
Tristichopterus	?0???	1110?	?0?01	1???0	00000	00?00	??010	00000	?0-00	?0???
Ventastega	10?-1	?0?02	?101?	?2011	100?0	10100	001-1	00111	00-21	11101
Whatcheeria	?????	?0002	01?1?	?2110	00210	20100	00100	10111	01021	11101
Youngolepis	01001	10010	10001	20-00	00?01	00000	??000	010A0	?0-00	00000

	5<br>1		6<br>1		7<br>1		8<br>1		9<br>1	
Acanthostega	00200	10100	00202	01011	00010	00012	00101	21011	11011	11000
Balanerpeton	01001	?0101	11200	0?110	10022	11110	1?121	20-12	00001	1100-
Baphetes	??000	?0101	11202	0?111	00020	11110	1?111	20-11	00001	1100-
Barameda	??10?	??1??	????0	????0	?0???	?????	?0100	00?1?	00000	?000?
Beelarongia	???0?	?????	?????	?????	?????	?????	?0???	10?1?	00?0?	001?0
Cabonnichthys	0?100	10100	00221	010?0	00000	00?01	00000	10010	00000	00100
Canowindra	???0?	?????	?????	?????	?????	?????	?00??	?0?1?	00000	00100
Cladarosymblema	00100	01100	00100	010?0	?0000	?????	?0010	10010	00000	00000
Crassigyrinus	?0001	?0100	10202	0?1?1	00010	11003	00111	21?12	00001	1100-
Dendrerpeton	0?00?	?0011	11200	??110	10022	1111?	10111	20-1?	00001	1100-
Diabolepis	?00?-	?00--	-1000	000?0	01?0?	-??-?	-?0??	?000-	1????	?0???
Dipterus	?0?--	?0?--	--20-	0?011	01?-?	-??-?	-00??	0000-	10?00	10000
Ectosteorhachis	00100	011?0	00100	010?0	?0?0?	?????	??010	10010	00000	0000?
Elginerpeton	??0??	?????	?????	?????	?????	?0???	?????	???1?	1????	?????
Elpistostege	??000	?????	?????	0????	?????	?????	?1000	00???	?0011	0101?
Eoherepton	1201?	?????	?????	1?11?	101?0	110?0	10121	21-1?	01011	1100-
Eusthenodon	?0100	10100	00221	???10	00?0?	000??	00000	10?10	00100	00100
Eusthenopteron	00000	00100	00221	01010	00000	00001	00000	10010	00000	00000
Glyptolepis	00000	10100	00000	00000	00000	10010	00000	0020-	00000	00000
Glyptopomus	??00?	00100	??2??	???10	0??00	???1?	??000	10?1?	00000	00000
Gogonasus	00000	001?0	00200	01000	00000	00010	00000	10010	00000	00000
Gooloogongia	???0?	0????	?????	?????	?0???	?????	?0100	00?1?	00001	00000
Greererepton	02200	??11	10??2	0?111	00011	1101?	00121	21?11	10001	1100-
Gyroptychius	00000	00100	00100	010?0	?0000	000?0	00000	10010	00000	00010
Ichthyostega	00201	10100	10202	01011	00100	1101B	0010?	20012	11011	11000
Jarvikina	?00?0	?010?	??221	?1??0	?0???	?????	??000	1001?	00000	000?0
Kenichthys	00000	0????	????0	010??	?0?0?	?00?0	00?00	?000-	00000	00001
Koharalepis	???0?	0?10?	?????	???10	?????	?????	?00??	?0?1?	00000	00100
Mandageria	??100	10100	00221	01010	00000	00001	00000	10010	00100	00110
Marsdenichthys	???0?	1010?	???0?	010?0	?00?0	0??0?	?0000	10?1?	00000	00?00
Medoevia	00100	00100	00100	01000	00000	00010	00???	?0?10	00000	00000
Megalichthys	00100	011?0	00100	01000	?0000	?00?0	00010	10010	00000	00000
Osteolepis	00000	0????	?????	010?0	00000	000?0	00000	10?10	00000	00000
Panderichthys	00000	10100	00211	01010	00000	00010	01000	00010	10000	110A0
Pederpes	???00	?????	????2	0???1	?00?0	11113	0???1	?????	?0001	11000
Platycephalichthys	0010?	101?0	002?1	?????	???00	?????	??000	10?10	00?00	0???1
Porolepis	00000	101??	??000	00000	00000	????0	?00??	0020-	00000	00000
Powichthys	?00?0	?0??0	00002	0000?	?0000	0????	??0??	0020-	0?0??	?0???
Proterogyrinus	12010	??0??	????2	1?111	100?0	11000	10121	20-1?	1?001	1100-
Silvanerpeton	02010	??001	11??2	0?111	10020	1??0?	?0121	20-1?	00001	1100-
Spodichthys	00?0?	00???	???2?	???10	000?0	0???0	?????	1??1?	0????	?0???
Tiktaalik	00000	1010?	0?211	01010	00000	00010	00?00	?001?	10011	11010
Tinirau	00000	10100	00221	01010	00000	00010	000--	10?10	00000	00??1
Tristichopterus	00000	0?100	00221	0?0?0	?0???	0????	?0???	10?10	00000	00000
Ventastega	00200	1?100	?0???	0?0?1	00000	010??	?01?1	21??1	?1011	11000
Whatcheeria	0020?	???01	10??2	??1?1	000?0	11000	00121	20?11	?1011	11000
Youngolepis	00000	001?-	-1000	000?0	?0?0?	001?0	0?0??	?010-	0?000	000?1

	101		111		121		131		141	
Acanthostega	00230	11100	-0100	1-110	0021-	-0000	01101	01013	--111	01101
Balanerpeton	00220	11000	00100	1-010	0021-	-????	1-101	0-113	--111	-2?1?
Baphetes	00230	11100	00100	1-010	0011-	-0000	03?01	0-113	--111	-2?1?
Barameda	??000	00000	00000	00001	00000	10001	?????	???0?	110??	????0
Beelarongia	00000	???00	00000	00000	11000	???0?	???0?	?0001	110??	??000
Cabonnichthys	00110	00001	00000	11000	00101	0000?	???00	00001	01000	00001
Canowindra	00100	???00	00000	10?00	11000	???0?	??00?	?000?	110??	000??
Cladarosymblema	00010	00000	00011	--000	00000	00001	00000	00001	01000	00000
Crassigyrinus	00230	11100	00100	1-010	0021-	-0000	03101	0-113	--111	-201?
Dendrerpeton	00230	11000	00100	1-010	0021-	-????	1-101	0-113	--111	-211?
Diabolepis	??001	????0	01011	0-?10	000??	?1110	?????	?????	?????	?????
Dipterus	0??11	0?000	01011	?-?10	0011-	-??01	00?0?	??00	00000	10001
Ectosteorhachis	0?010	????0	00011	--000	00???	?0001	00???	?????	010??	?????
Elginerpeton	????0	?????	?????	?????	?????	?????	000??	?????	?????	???1?
Elpistostege	??23?	10110	??1??	1-1?0	0?21-	-????	00??0	1????	?????	?????
Eoherepton	00230	01?00	00100	1-010	0021-	-????	1-101	0-11?	?????	?????
Eusthenodon	00110	00001	10000	11000	00101	0000?	??00	0000?	01000	0000?
Eusthenopteron	00110	00000	00000	00000	00101	00001	00000	00001	01000	00001
Glyptolepis	11000	??00	??011	0-000	1-000	00101	00000	00000	00000	10000
Glyptopomus	00100	00000	00000	00000	00000	0??0?	0???0	00001	0100?	????1
Gogonasus	00100	00000	00000	0-000	00000	00001	00000	00002	01000	000?0
Gooloogongia	00100	00000	00000	00001	00000	1000?	??00	00000	11000	100??
Greererepton	00220	10110	-0100	1-?10	0011-	-0000	01101	0-11?	-?111	-210?
Gyroptychius	00100	0??00	00000	10000	00000	00001	00000	00001	01000	00001
Ichthyostega	00230	11100	-0100	0-110	0021-	-0000	02101	01013	--111	-2101
Jarvikina	00110	????1	00000	10?00	001??	???0?	???0?	?????	010??	?????
Kenichthys	00000	??00	?0?10	0-?00	00000	01101	00?1?	?????	010??	????0
Koharalepis	00000	0?000	00000	10000	11000	??01	00000	00001	11000	??0??
Mandageria	00110	00001	10000	11000	01101	?000?	??00	0000?	11000	00001
Marsdenichthys	00110	????0	00000	00000	10000	0????	????0	?0002	11000	??0??
Medoevia	00010	0?110	00000	00000	01000	?????	????0	00001	01000	0?000
Megalichthys	00??0	??00	00011	--000	000??	?0001	??00	00001	010??	000?0
Osteolepis	00110	0?000	00000	00000	00000	00001	0000?	?0001	01000	00000
Panderichthys	00220	00010	00100	1-010	0011-	-0001	00000	1?002	01000	00101
Pederpes	00??0	01?0?	??1??	??0??	?011-	-????	???0?	?1013	--111	-211?
Platycephalichthys	??1?0	00???	??000	--0??	?????	?0?0?	0?0??	?00??	?????	?????
Porolepis	11000	????0	?1011	0-000	1-100	00101	00000	00001	00000	10000
Powichthys	?1000	????0	01010	10010	001??	?1101	?????	?????	00000	????0
Proterogyrinus	00230	11000	00100	0-010	0021-	-????	1-101	0-113	--111	-201?
Silvanerpeton	00230	01000	00100	0-010	0021-	-????	1-101	0-113	--111	-201?
Spodichthys	??100	???0?	00000	00000	00100	00?0?	0?000	???0?	??0??	??001
Tiktaalik	00230	1?1?0	-0100	0-?10	0011-	-??00	00000	10013	--110	00101
Tinirau	00100	0000?	?0000	1-000	00100	00001	00000	00001	0?0??	??001
Tristichopterus	00110	00000	00000	0-000	00101	???0?	?0?00	00001	010??	00001
Ventastega	00230	1??00	001??	1-010	0?11-	?0???	0000?	??013	-?111	01?11
Whatcheeria	00230	??00	00100	0-010	0011-	-????	01001	01013	--111	-2?0?
Youngolepis	00000	?????	?1?11	0-010	001??	?1110	???1?	?????	?????	????0

	151		161		171		181		191	
Acanthostega	21011	11110	11111	00111	00???	0011-	10102	1001-	1-111	11-01
Balanerpeton	21?11	1?110	111??	11111	110?0	0011-	10102	1001-	1-??1	11-?1
Baphetes	??11	1???0	11110	11111	11???	?????	?????	?001-	????1	11?01
Barameda	???1?	????0	0010?	00001	00010	?0001	?????	?????	????0	0??1?
Beelarongia	?????	?????	001??	?????	?????	??00	?????	?????	????0	00?0?
Cabonnichthys	???1?	????0	0010?	00000	00001	01000	?????	?????	0?100	00110
Canowindra	?????	?????	?????	?????	?????	?????	?????	?????	0?0?0	0?01?
Cladarosymblema	??010	0????	?????	?????	?????	??00	?????	?????	????3	00?00
Crassigyrinus	???1?	??110	11111	11111	11???	0011-	10110	1??1-	1-??2	11-?1
Dendrerpeton	21211	1?110	1111?	11111	11000	0011-	10110	1001-	1-??1	11-?1
Diabolepis	?????	?????	?????	?????	?????	?????	?????	?????	????3	0????
Dipterus	???0?	????1	?????	?????	?????	?0001	00100	0????	010?3	000?0
Ectosteorhachis	?????	?????	?????	?????	?????	??00	?????	?????	????3	0??0?
Elginerpeton	??01?	?????	111??	?????	?????	?????	?????	?11??	????1	11???
Elpistostege	?????	?????	?????	?????	?????	?????	?????	?????	????0	?????
Eoherepton	21211	11??0	111-0	1111?	1????	?????	10110	1001-	1-??1	0?-??
Eusthenodon	?????	?????	?????	?????	?????	?????	?????	?????	0???0	00?10
Eusthenopteron	00010	00000	00100	00000	00001	01000	00000	10-00	00100	00110
Glyptolepis	00000	0?0?1	000??	?????	?????	?0001	00000	0????	01003	00010
Glyptopomus	?????	?????	?????	?????	?????	??00	?????	?????	0?100	0?000
Gogonasus	???1?	????0	01100	00000	000?1	01000	?????	?????	????3	00?0?
Gooloogongia	?0???	??0?0	?????	00000	00010	??01	00???	??00	000?0	00010
Greererepton	21111	11110	11111	11111	11???	0011-	10111	1001-	1-??1	11-01
Gyroptychius	?????	?????	?????	?????	?????	??00	00000	1????	0?103	00100
Ichthyostega	21010	11120	11111	11111	11???	0011-	11111	1111-	1-111	11-??
Jarvikina	?????	?????	?????	?????	?????	?????	?????	?????	????0	0??1?
Kenichthys	?????	?????	?????	?????	?????	?????	?????	?????	????3	0?10?
Koharalepis	?????	?????	?????	?????	?????	??00	?????	?????	????0	00?0?
Mandageria	???1?	????0	001?0	00000	00001	01000	?????	?????	00100	00010
Marsdenichthys	?????	?????	?????	?????	?????	?????	?????	?????	0???0	00?10
Medoevia	00010	0000?	00100	?0???	?????	?????	?????	?????	0???3	00100
Megalichthys	00010	0?0?0	00100	00000	00001	01000	-----	1????	00003	00100
Osteolepis	?????	????0	?????	?????	?????	??00	-----	1????	00003	00100
Panderichthys	11011	110?0	01100	00100	00000	0?000	00101	1??01	1-1?0	00-00
Pederpes	21111	1?120	1111?	11111	11???	?011-	11111	1011-	1-??1	11-?1
Platycephalichthys	00???	0????	?????	?????	?????	?????	?????	?????	????0	0??0?
Porolepis	?????	?????	?????	?????	?????	?????	?????	?????	??0?3	00?00
Powichthys	?????	?????	?????	?????	?????	?????	?????	?????	????3	0??0?
Proterogyrinus	21211	11110	11110	11111	11???	0011-	10110	1001-	1-??1	11-01
Silvanerpeton	21211	1?120	111??	11111	11???	0011-	10110	1011-	1-??1	11-?1
Spodichthys	000??	?????	?????	?????	?????	?????	?????	?????	????0	00???
Tiktaalik	11011	1?0?0	01101	01101	00110	10001	11?01	?0-??	1-??0	00-00
Tinirau	00?10	01??0	?????	0??0?	??001	01000	00000	10-01	0?000	00?10
Tristichopterus	???1?	????0	00100	00000	00001	01000	?????	1????	00000	00110
Ventastega	210??	??110	?????	?????	?????	?????	?????	?00??	????1	11???
Whatcheeria	2121?	??120	111?1	?11??	1????	?????	11111	101??	1-??3	11-??
Youngolepis	000?0	0????	?????	?????	?????	?????	?????	?????	????3	0??0?

	**2**<br>**0**<br>**1**
Acanthostega	2102
Balanerpeton	2102
Baphetes	210?
Barameda	?1??
Beelarongia	?00?
Cabonnichthys	110?
Canowindra	?1??
Cladarosymblema	100?
Crassigyrinus	210?
Dendrerpeton	?10?
Diabolepis	?01?
Dipterus	?01?
Ectosteorhachis	?00?
Elginerpeton	????
Elpistostege	????
Eoherepton	?10?
Eusthenodon	110?
Eusthenopteron	1100
Glyptolepis	31?0
Glyptopomus	?10?
Gogonasus	1001
Gooloogongia	?1??
Greererepton	2102
Gyroptychius	1000
Ichthyostega	2102
Jarvikina	11??
Kenichthys	100?
Koharalepis	100?
Mandageria	110?
Marsdenichthys	?1??
Medoevia	?001
Megalichthys	100?
Osteolepis	100?
Panderichthys	210-
Pederpes	?10?
Platycephalichthys	?1??
Porolepis	3000
Powichthys	100?
Proterogyrinus	210?
Silvanerpeton	?10?
Spodichthys	????
Tiktaalik	2102
Tinirau	?100
Tristichopterus	?10?
Ventastega	????
Whatcheeria	?10?
Youngolepis	100?

Character Optimizations

Rhizodonts + other tetrapodomorphs:
- 89, 0→1 = one pair of external nostrils
- 114, 1→0 = parietals surround a parietal foramen/eminance
- 127, 1→0 = premaxilla is canal bearing
- 128, 1→0 = infraorbital canal does not follow the premaxillary suture
- 195, 3→0 = tuberculate ornament
- 199, 0→1 = round body scales
- 202, 0→1 = loss of cosmine

Rhizodonts:
- 26, 0→1 = 1 pair of dentary fangs
- 83, 0→1 = 1 or 2 nasal bones
- 120, 0→1 = postparietals narrow to a point posteriorly
- 126, 0→1 = contact between postspiracular and supratemporal

'Osteolepiforms' + elpistostegalians:
- 86, 0→1 = median postrostral present
- 140, 0→1 = small opening to spiracular notch
- 146, 1→0 = exposed anocleithrum
- 180, 1→0 = basial lepidotrichial segments not elongate

Canowindrids:
- 121, 0→1 = PP shield extremely wide posteriorly

Canowindrids (minus *Marsdenichthys*):
- 122, 0→1 = supratemporal fused with postparietals

Canowindrids (*Koharalepis* + *Beelarongia* only):
- 103, 1→0 = width of ethmoid ≥80%
- 199, 1→0 = rhomboid body scales
- 202, 1→0 = cosmine present

Megalichthyiforms + eotetrapodiforms:
- 198, 0→1 = basal scutes present

Megalichthyiforms:
- 15, 1→0 = dorsal directly above ventral hyomandibular facet
- 69, 1→0 = anterior end of entopterygoid level with processus ascendens
- 195, 0→3 = ornament absent or almost absent
- 199, 1→0 = rhomboid body scales
- 202, 1→0 = cosmine present
- 204, 0→1 = hyomandibulae orientation almost horizontal, opercular process high up dorsally

Megalichthyiforms (minus *Gogonasus*):
- 63, 2→1 = anteromedial process of vomer present

Megalichthyiforms (minus *Gyroptychius*):
- 104, 0→1 = 33-40% of skull roof lies anterior to orbits

Osteolepidids (*Medoevia* + megalichthyids):
- 53, 0→1 = enlarged premaxillary tooth
- 103, 1→0 = width of ethmoid ≥80%

Megalichthyiforms (megalichthyids only)
- 26, 0→1 = 1 pair of dentary fangs
- 57, 0→1 = vomers much broader than long
- 114, 0→1 = parietals do not surround the pineal foramen

- 115, 0→1 = pineal foramen absent

Eotetrapodiforms:

- 64, 0→2 = long posterior processes on vomers
- 65, 0→1 = overlap of vomers and parasphenoid
- 123, 0→1 = posterior margin of tabular level with posterior margin of postparietals
- 150, 0→1 = contact margin for clavicle on cleithrum strongly concave

Tristichopterids:

- 16, 0→1 = parasymphyseal plate short not sutured to coronoid
- 34, 0→1 = Posterior coronoid longer than more anterior coronoids

Tristichopterids (minus *Spodichthys*):

- 104, 0→1 = 33-40% of skull roof anterior to orbits
- 125, 0→1 = posteriorly displaced PSP

Tristichopterids (*Eusthenopteron* + remaining tristichopterids):

- 35, 0→1 = posterior coronoid one-third longer than more anterior coronoids
- 44, 0→1 = 2 fang pairs on posteriormost coronoid
- 193, 0→1 = diphycercal caudal fin

Tristichopterids (*Jarvikina* + remaining tristichopterids):

- 110, 0→1 = posterior orbital process much longer than orbital margin
- 116, 0→1 = pineal foramen well posterior to orbital margin

Tristichopterids (*Cabonnichthys* + remaining tristichopterids):

- 26, 0→1 = 1 pair of dentary fangs
- 53, 0→1 = enlarged premaxillary tooth
- 98, 0→1 = postorbital excluded from orbital margin
- 117, 0→1 = pineal series kite-shaped

Tristichopterids (*Mandageria* + *Eusthenodon* only):

- 93, 0→1 = contact between lacrimal and posterior supraorbital
- 111, 0→1 = no contact between intertemporal and posterior supraorbital

Tinirau + [*Platycephalichthys* + Elpistostegalia]:

- 26, 0→1 = 1 pair of dentary fangs
- 35, 0→1 = posterior coronoid one-third longer than more anterior coronoids
- 45, 0→1 = organized tooth row on posterior coronoid
- 56, 0→1 = posterodorsal maxillary process weak/absent
- 116, 0→1 = pineal foramen posterior to orbits
- 157, 0→1 = height/width ratio of glenoid fossa, 40-50%
- 190, 0→1 = highly reduced postaxial process on fibula

Platycephalichthys + Elpistostegalia:

- 4, 0→1 = In posterior view, the fenestra ventrolateralis extends dorsal to the ethmoid articulation
- 199, 1→0 = rhomboid scales

Elpistostegalia:

- 48, 0→1 = prearticular contacts angular edge-to-edge
- 86, 1→0 = median postrostral absent
- 91, 0→1 = anterior nostril at edge of mouth
- 96, 0→1 = jugal/quadratojugal contact
- 103, 1→2 = ethmoid proportions ≤35%
- 113, 0→1 = frontals present
- 151, 0→1 = scapulocoracoid, large plate pierced by coracoid foramen
- 152, 0→1 = coracoid plate present
- 156, 0→1 = lateral component to glenoid orientation

Elpistostegalia minus *Panderichthys*:

- 44, 0→2 = no fang pairs on posterior-most coronoid
- 94, 0→1 = jugal extends anterior to front of orbit
- 95, 0→1 = jugal extends anterior to middle of orbit
- 106, 0→1 = anterior supraorbital 3x longer than broad
- 108, 0→1 = prefrontal much bigger than postfrontal
- 130, 1→0 = no fusion of supra and infraorbital canals
- 139, 0→1 = loss of opercular
- 143, 0→1 = loss of extrascapular bones
- 144, 0→1 = lost of posttemporals
- 165, 0→1 = ectepicondytlar processes extends distal to epipodial facets
- 170, 0→1 = radius is shorter than the humerus
- 181, 0→1 = expanded ribs present
- 204: 0→2 = very short, laterally directed hyomandibulae

Elpistostege + *Tiktaalik*:

- 99, 0→1 = contact between postorbital and lacrimal

Elginerpeton + remaining elpistostegalians:

- 21, 0→1 = mesial parasymphyseal foramen present
- 33, 0→1 = loss of Meckelian exposure in precoronoid fossa
- 46, 0→1 = forked prearticular
- 47, 0→1 = prearticular sutured to mesial lamina of splenial (i.e., mesial lamina of the splenial present)
- 50, 0→1 = well-defined dorsal longitudinal band of shagreen on prearticuular
- 161, 0→1 = square/parallelogram-shaped entepicondyle on humerus
- 195, 0→1 = fairly regular pit and ridge derma ornament
- 196, 0→1 = starbursts radiating on at least some bones
- 197, 0→1 = loss of cleithral ornamentation

Ventastega + remaining elpistostegalians:

- 39, 0→1 = anterior and middle coronoid teeth ≤½ the height of dentary teeth
- 40, 0→1 = anterior coronoid contacts splenial
- 53, 0→2 = posterior teeth ≥2x height of anterior teeth

Acanthostega + remaining elpistostegalians:

- 13, 0→1 = single large foramen in the hypophyseal region of braincase
- 35, 1→0 = posterior coronoid not substantially longer than anterior coronoids
- 132, 0→1 = mandibular line canal mostly enclosed but short sections with open grooves
- 133, 0→1 = no surangular pit line

Ichthyostega + remaining elpistostegalians:

- 23, 0→2 = dentary teeth smaller tan maxillary teeth
- 61, 0→1 = anterior wall of vomer lacks teeth along the ridge
- 76, 0→1 = medial exposure of dermopalatine, in addition to tooth roow
- 147, 1→2 = loss of anocleithrum
- 159, 1→2 = interclavicle with a posterior stalk
- 166, 0→1 = radius and ulna about equal in length
- 171, 0→1 = ulnar facet has some ventrally directed component
- 172, 0→1 = olecranon process present
- 184, 0→1 = at least some ventral component to ribs

Whatcheeria + remaining elpistostegalians:

- 21, 1→0 = no mesial parasymphyseal foramen

- 26, 1→2 = 1 unpaired dentary fang (i.e., no replacement pit)
- 42, 0→1 = posterodorsal process of posterior coronoid
- 60, 0→1 = no row of small teeth on the vomer
- 68, 0→1 = parasphenoid overlaps basioccipital
- 84, 0→2 = loss of anterior tectal

Pederpes + remaining elpistostegalians:

- 92, 1→0 = lacrimal contributes to orbital margin
- 94, 1→0 = jugal does not extend anterior to the anterior orbital margin

Greererpeton + remaining elpistostegalians:

- 138, 0→1 = loss of preoperculum
- 159, 2→1 = kite-shaped interclavicle (i.e., no posterior stalk)
- 182, 1→0 = loss of imbricate ribs
- 188, 1→0 = posterior process on ileum oriented posterodorsally

Crassigyrinus + remaining elpistostegalians:

- 53, 2→0 = all premaxillary teeth all the same size
- 91, 1→0 = facial position of anterior external nostril (not edge of mouth)
- 132, 1→3 = entirely open mandibular line canal
- 185, 1→0 = all ribs cylindrical

Baphetes + remaining elpistostegalians:

- 62, 0→1 = vomerine shagreen field present
- 74, 1→2 = anterior palatal fanestra absent
- 81, 0→1 = no row of 3+ smaller teeth on ectopterygoid
- 165, 1→0 = ectepicondylar process terminates proximal to epipodial facets

Stem-lissamphibians + stem-amniotes + embolomeres:

- 71, 0→1 = convex component to the ectopterygoid/quadratojugal in the subtemporal fossa
- 108, 1→0 = anterior and posterior surpraorbitals of similar size
- 131, 0→1 = loss of mandibular sensory line canal

Stem-lissamphibians (*Balanerpeton* + *Dendrerpeton*):

- 65, 2→0 = no contact (via gap – or simple abutment) between vomers and parasphenoid
- 70, 1→0 = entopterygoids do not meet at midline
- 75, 0→2 = interentopterygoid vacuities <2x longer than wide

Stem-amniotes (*Sylvanerpeton* + embolomeres):

- 23, 1→0 = De teeth same size as Mx teeth
- 54, 0→1 = Mx does not extend behind posterior orbital margin
- 79, 1→0 = ectopterygoid does not reach subtemporal fossa

Embolomeres (*Proterogyrinus* + *Eoherpeton*):

- 51, 0→1 = Prearticular with mesially projecting flange on dorsal edge along posterior border of adductor fossa
- 66, 0→1 = denticulated field of parasphenoid absent

References

1. Ahlberg PE, Clack JA, Lukševičs E, Blom H, Zupiņš I (2008) *Ventastega curonica* and the origin of tetrapod morphology. Nature 453: 1199—1204.
2. Ahlberg PE, Johanson Z (1998) Osteolepiforms and the ancestry of tetrapods. Nature 395: 792—793.
3. Ahlberg PE, Lukševičs E, Mark-Kurik E (2000) A near-tetrapod from the Baltic Middle Devonian. Palaeontology 43: 533—548.
4. Coates MI, Friedman M (2010) *Litoptychus bryanti* and characteristics of stem tetrapod neurocrania. In: Elliott DK, Maisey JG, Yu X, Miao D, editors. Morphology, Phylogeny and Paleobiogeography of Fossil Fishes. München: Verlag Dr. Friedrich Pfeil. pp. 389—416.
5. Daeschler EB, Shubin NH, Jenkins Jr FA (2006) A Devonian tetrapod-like fish and the evolution of the tetrapod body plan. Nature 440: 757—763.
6. Zhu M, Ahlberg PE (2004) The origin of the internal nostril of tetrapods. Nature 432: 94—97.
7. Long JA, Young GC, Holland T, Senden TJ, Fitzgerald EMG (2006) An exceptional Devonian fish from Australia sheds light on tetrapod origins. Nature 444: 199—202.
8. Ahlberg PE, Clack JA (1998) Lower jaws, lower tetrapods—a review based on the Devonian genus *Acanthostega*. Transactions of the Royal Society of Edinburgh: Earth Sciences 89: 11—46.
9. Clack JA (1988) New material of the early tetrapod *Acanthostega* from the Upper Devonian of East Greenland. Palaeontology 31: 699—724.
10. Clack JA (1989) Discovery of the earliest-known tetrapod stapes. Nature 432: 425—427.
11. Clack JA (1994) *Acanthostega gunnari*, a Devonian tetrapod from Greenland; the snout, palate and ventral parts of the braincase, with a discussion of their significance. Meddelelser om Gronland Geoscience 31: 1—24.
12. Clack JA (1998) The neurocranium of *Acanthostega gunnari* Jarvik and the evolution of the otic region in tetrapods. Zoological Journal of the Linnean Sociely 122: 61—97.
13. Coates MI (1996) The Devonian tetrapod *Acanthostega gunnari* Jarvik: postcranial anatomy, basal tetrapod interrelationships and patterns of skeletal evolution. Transactions of the Royal Society of Edinburgh: Earth Sciences 87: 363—421.
14. Clack JA (2002) The dermal skull roof of *Acanthostega gunnari*, an early tetrapod from the Late Devonian. Transactions of the Royal Society of Edinburgh: Earth Sciences 93: 17—33.
15. Milner A, Sequeira S (1993) The temnospondyl amphibians from the Viséan of East Kirkton, West Lothian, Scotland. Transactions of the Royal Society of Edinburgh: Earth sciences 84: 331—361.
16. Beaumont EH (1977) Cranial morphology of the Loxommatidae Amphibia Labyrinthodontia. Philosophical Transactions of the Royal Society of London B Biological Sciences 280: 29—101.
17. Milner AC, Lindsay W (1998) Postcranial remains of *Baphetes* and their bearing on the relationships of the Baphetidae (= Loxommatidae). Zoological Journal of the Linnean Sociely 122: 211—235.
18. Owen R (1854) On some fossil reptilian and mammalian remains from the Purbecks. Quarterly Journal of the Geological Society of London 10: 420—433.
19. Watson DMS (1929) The Carboniferous Amphibia of Scotland. Palaeontologica Hungarica 1: 219—252.
20. Garvey JM, Johanson, Z. and Warren, A. (2005) Redescription of the pectoral fin and vertebral column of the rhizodontid fish *Barameda decipiens* from the Lower Carboniferous of Australia. Journal of Vertebrate Paleontology 25: 8—18.
21. Long JA (1989) A new rhizodontiform fish from the Early Carboniferous of Victoria, Australia, with remarks on the phylogenetic position of the group. Journal of Vertebrate Paleontology 9: 1—17.
22. Long JA, Ahlberg PE (1999) New observations on the snouts of rhizodont fishes (Palaeozoic Sarcopterygii). Records of the Australian Museum Supplements 57: 163—173.

23. Long JA (1987) An unusual osteolepiform fish from the Late Devonian of Victoria, Australia. Palaeontology 30: 839—852.
24. Ahlberg PE, Johanson Z (1997) Second tristichopterid (Sarcopterygii, Osteolepiformes) from the Upper Devonian of Canowindra, New South Wales, Australia, and phylogeny of the Tristichopteridae. Journal of Vertebrate Paleontology 17: 653—673.
25. Thomson KS (1973) Observations on a new rhipidistian fish from the Upper Devonian of Australia. Palaeontographica Abteilung A 143: 209—220.
26. Long J (1985) New information on the head and shoulder girdle of *Canowindra grossi* Thomson, from the Late Devonian Mandagery Sandstone, New South Wales Australia. Records of the Australian Museum 37: 91—100.
27. Fox RC, Campbell KSW, Barwick RE, Long JA (1995) A new osteolepiform fish from the Lower Carboniferous Raymond Formation, Drummond Basin, Queensland. Memoirs of the Queensland Museum 38: 97—221.
28. Panchen A, Smithson T (1990) The pelvic girdle and hind limb of *Crassigyrinus scoticus* (Lydekker) from the Scottish Carboniferous and the origin of the tetrapod pelvic skeleton. Transactions of the Royal Society of Edinburgh: Earth Sciences 81: 31—44.
29. Clack JA (1998) The Scottish Carboniferous tetrapod *Crassigyrinus scoticus* (Lydekker)—cranial anatomy and relationships. Transactions of the Royal Society of Edinburgh: Earth Sciences 88: 127—142.
30. Godfrey SJ, Fioriollo AR, Carroll RL (1987) A newly discovered skull of the temnospondyl amphibian *Dendrerpeton acadianum* Owen. Canadian Journal of Earth Sciences 24: 796—805.
31. Holmes RB, Carroll RL, Reisz RR (1998) The first articulated skeleton of *Dendrerpeton acadianum* (Temnospondyli, Dendrerpetontidae) from the Lower Pennsylvanian locality of Joggins, Nova Scotia, and a review of its relationships. Journal of Vertebrate Paleontology 18: 64—79.
32. Owen R (1853) Notes on the above-described fossil remains. Quarterly Journal of the Geological Society 9: 66—67.
33. Robinson J, Ahlberg PE, Koentges G (2005) The braincase and middle ear region of *Dendrerpeton acadianum* (Tetrapoda: Temnospondyli). Zoological Journal of the Linnean Society 143: 577—597.
34. Chang M-m (1995) *Diabolepis* and its bearing upon the relationships between porolepiforms and dipnoans. Bulletin du Muséum d'Histoire naturelle, Paris 17: 235—268.
35. Chang M-m, Yu X (1984) Structure and phylogenetic significance of *Diabolichthys speratus* gen. et sp. nov., a new dipnoan-like form from the Lower Devonian of Eastern Yunnan, China. Proceedings of the Linnean Society of New South Wales 107: 171—184.
36. Smith MM, Chang M-m (1990) The dentition of *Diabolepis speratus* Chang and Yu, with further consideration of its relationships and the primitive dipnoan dentition. Journal of Vertebrate Paleontology 10: 420—433.
37. Ahlberg PE, Trewin NH (1995) The postcranial skeleton of the Middle Devonian lungfish *Dipterus valenciennesi*. Transactions of the Royal Society of Edinburgh: Earth Sciences 85: 159—175.
38. White EI (1965) The head of *Dipterus valenciennes* Siedgwick and Murchison. Bulletin of the British Museum (Natural History) 11: 1—45.
39. Thomson KS (1964) Revised generic diagnoses of the fossil fishes *Megalichthys* and *Ectosteorhachis* (Family Osteolepidae). Bulletin of the Museum of Comparative Zoology 131: 283—311.
40. Ahlberg PE (1991) Tetrapod or near-tetrapod fossils from the Upper Devonian of Scotland. Nature 354: 298—301.
41. Ahlberg PE (1995) *Elginerpeton pancheni* and the earliest tetrapod clade. Nature 373: 420—425.
42. Ahlberg PE (1998) Postcranial stem tetrapod remains from the Devonian of Scat Craig, Morayshire, Scotland. Zoological Journal of the Linnean Sociely 122: 99—141.

43. Schultze H-P, Arsenault M (1985) The panderichthyid fish *Elpistostege*—a close relative of tetrapods. Palaeontology 28: 293—309.
44. Andrews SM, Browne MAE, Panchen AL, Wood SP (1977) Discovery of amphibians in the Namurian (Upper Carboniferous) of Fife. Nature 265: 529—532.
45. Smithson TR (1985) The morphology and relationships of the Carboniferous amphibian *Eoherpeton watsoni*. Zoological Journal of the Linnean Society 85: 317—410.
46. Jarvik E (1952) On the fish-like tail in the ichthyostegid stegocephalians with descriptions of a new stegocephalian and a new crossopterygian from the upper Devonian of East Greenland. Meddelelser om Grønland 114: 5—90.
47. Andrews SM, Westoll TS (1970) The postcranial skeleton of *Eusthenopteron foordi*. Transactions of the Royal Society of Edinburgh 68: 207—329.
48. Jarvik E (1980) Basic Structure and Evolution of Vertebrates, Volume 1. London: Academic Press. 575 p.
49. Ahlberg P (1989) Paired fin skeletons and relationships of the fossil group Porolepiformes (Osteichthyes: Sardcopterygii). Zoological Joural of the Linnean Society 96: 119—166.
50. Ahlberg PE (1991) A re-examination of sarcopterygian interrelationships, with special reference to the Porolepiformes. Zoological Journal of the Linnean Society 103: 241—287.
51. Jarvik E (1972) Middle and Upper Devonian Porolepiformes from East Greenland with special reference to *Glyptolepis groenlandica* n.sp. Meddelelser om Grønland 182: 1—307.
52. Jarvik E (1950) Middle Devonian vertebrates from Canning Land and Wegeners Halvö (East Greenland). Part II. Crossopterygii. Meddelelser om Grønland 96: 1—132.
53. Long JA, Barwick RE, Campbell KSW (1997) Osteology and functional morphology of the osteolepiform fish *Gogonasus andrewsae* Long, 1985, from the Upper Devonian Gogo Formation, Western Australia. Records of the Australian Museum Supplements 53: 1—89.
54. Johanson Z, Ahlberg PE (2001) Devonian rhizodontids and tristichopterids (Sarcopterygii; Tetrapodomorpha) from East Gondwana. Transactions of the Royal Society of Edinburgh: Earth Sciences 92: 43—74.
55. Bolt JR, Lombard E (2001) The mandible of the primitive tetrapod *Greererpeton*, and the early evolution of the tetrapod lower jaw. Journal of Paleontology 75: 1016—1042.
56. Godfrey SJ (1989) The postcranial skeletal anatomy of the Carboniferous tetrapod *Greererpeton burkemorani* Romer 1969. Philosophical Transactions of the Royal Society of London B Biological Sciences 323: 75—134.
57. Romer AS (1969) A temnospondylous labyrinthodont from the lower Carboniferous. Kirtlandia No. 6: 1—20.
58. Smithson TR (1982) The cranial morphology of *Greererpeton burkemorani* Romer (Amphibia: Temnospondyli). Zoological Journal of the Linnean Sociely 76: 29—90.
59. Jarvik E (1950) On some osteolepiform crossopterygians from the Upper Old Red Sandstone of Scotland. Kungl Svenska Vetenskapsakademiens Handlingar, series 4 2: 1—35.
60. Jarvik E (1950) Note on Middle Devonian crossopterygians from the eastern part of Gauss Halvö, East Grenland. Meddelelser om Grønland 149: 1—20.
61. Jarvik E (1985) Devonian osteolepiform fishes from East Greenland. Meddelelser om Grønland 13: 1—52.
62. Ahlberg PE, Clack JA, Blom H (2005) The axial skeleton of the Devonian tetrapod *Ichthyostega*. Nature 437: 137—140.
63. Jarvik E (1996) The Devonian tetrapod *Ichthyostega*. Fossils and Strata 40: 1—213.
64. Vorobyeva EI (1977) Morphology and nature of evolution of crossopterygian fishes. Trudy Paleontologischeskogo Instituta, Akademia Nauk SSSR 163: 1—239.
65. Chang M-m, Zhu M (1993) A new Middle Devonian osteolepidid from Quijing, Yunnan. Memoirs of the Association of Australasian Palaeontologists 15: 183—198.

66. Young GC, Long JA, Ritchie A (1992) Crossopterygian fishes from the Devonian of Antarctica: systematics, relationships, and biogeographic significance. Records of the Australian Museum Supplement: 1—77.
67. Johanson Z, Ahlberg PE (1997) A new tristichopterid (Osteolepiformes: Sarcopterygii) from the Mandagery Sandstone (Late Devonian, Famennian) near Canowindra, NSW, Australia. Transactions of the Royal Society of Edinburgh: Earth Sciences 88: 39—68.
68. Johanson Z, Ahlberg PE, Ritchie A (2003) The braincase and palate of the tetrapodomorph sarcopterygian *Mandageria fairfaxi*: morphological variability near the fish-tetrapod transition. Palaeontology 46: 271—293.
69. Holland T, Long J, Snitting D (2010) New information on the enigmatic tetrapodomorph fish *Marsdenichthys longioccipitus* (Long, 1985). Journal of Vertebrate Paleontology 30: 68—77.
70. Long JA (1985) The structure and relationships of a new osteolepiform fish from the Late Devonian of Victoria, Australia. Alcheringa: An Australasian Journal of Palaeontology 9: 1—22.
71. Lebedev OA (1995) Morphology of a new osteolepidid fish from Russia. Bulletin du Museum National d'Histoire Naturelle Section C Sciences de la Terre Paleontologie Geologie Mineralogie 17: 287—341.
72. Jarvik E (1948) On the morphology and taxonomy of the Middle Devonian osteolepid fishes of Scotland. K Svenska Vetenskapsakad Handl 25: 1—301.
73. Jarvik E (1967) Remarks on the structure of the snout in *Megalichthys* and certain other rhipidistian crossopterygians. Arkiv for Zoologi 19: 41—98.
74. Romer AS (1937) The braincase of the Carboniferous crossopterygian *Megalichthys nitidus*. Bulletin of the Museum of Comparative Zoology 82: 1—73.
75. Andrews SM, Westoll TS (1970) The postcranial skeleton of rhipidistian fishes excluding *Eusthenopteron*. Transactions of the Royal Society of Edinburgh: Earth Sciences 68: 391—489.
76. Watson DMS (1926) Croonian lecture: the evolution and origin of the Amphibia. Philosophical Transactions of the Royal Society of London B Biological Sciences 214: 189—257.
77. Thomson K (1965) The endocranium and associated structures in the Middle Devonian rhipidistian fish *Osteolepis*. Proceedings of the Linnean Society of London 176: 181—195.
78. Ahlberg PE, Clack JA, Lukševičs E (1996) Rapid braincase evolution between *Panderichthys* and the earliest tetrapods. Nature 381: 61—64.
79. Boisvert CA (2005) The pelvic fin and girdle of *Panderichthys* and the origin of tetrapod locomotion. Nature 438: 1145—1147.
80. Boisvert CA, Mark-Kurik E, Ahlberg PE (2008) The pectoral fin of *Panderichthys* and the origin of digits. Nature 456: 636—638.
81. Brazeau MD, Ahlberg PE (2006) Tetrapod-like middle ear architecture in a Devonian fish. Nature 439: 318—321.
82. Vorobyeva EI (1995) The shoulder girdle of *Panderichthys rhombolepis* (Gross) (Crossopterygii), Upper Devonian, Latvia. Geobios, MS 19: 285—288.
83. Vorobyeva EI (2000) Morphology of the humerus in the rhipidistian crossopterygii and the origin of tetrapods. Paleontologicheskii Zhurnal: 49—59.
84. Vorobyeva EI, Schultze H-P (1991) Description and systematics of panderichthyid fishes with comments on their relationship to tetrapods. In: Schultze H-P, Trueb L, editors. Origins of the Higher Groups of Tetrapods: Controversy and Consensus. Ithaca: Cornell University Press. pp. 68—109.
85. Clack JA (2002) An early tetrapod from 'Romer's Gap'. Nature 418: 72—76.
86. Clack JA, Finney SM (2005) *Pederpes finneyae*, an articulated tetrapod from the Tournaisian of Western Scotland. Journal of Systematic Palaeontology 2: 311—346.
87. Vorobyeva EI (1962) Rhizodont crossopterygian fishes from the Main Devonian Field of the USSR. Trudy Paleontologicheskogo Instituta 94: 1—139.

88. Clément G (2004) Nouvelles données anatomiques et morphologie générale des «Porolepidae» (Dipnomorpha, Sarcopterygii). Revue Paléobiology, Genève 9: 193—211.
89. Clément G, Janvier P (2004) *Powichthys spitsbergensis* sp. nov., a new member of the Dipnomorpha (Sarcopterygii, lobe-finned fishes) from the Lower Devonian of Spitsbergen, with remarks on basal dipnomorph anatomy. Fossils and Strata 50: 92—112.
90. Jessen HL (1975) A new choanate fish, *Powichthys torsteinssoni* n.g., n.sp., from the early Lower Devonian of the Canadian arctic archipelago. Problèmes actuels de paléontologie-évolution des vertebrés. Coll int CNRS 218: 213—225.
91. Jessen HL (1980) Lower Devonian Porolepiformes from the Canadian Arctic with special reference to *Powichthys thorsteinssoni*. Palaeontographica Abteilung A Palaeozoologie-Stratigraphie 167: 180-214.
92. Holmes R (1984) The Carboniferous amphibian *Proterogyrinus scheelei* and the early evolution of tetrapods. Philosophical Transactions of the Royal Society of London B Biological Sciences 306: 431—524.
93. Romer AS (1970) A new anthracosaurian labyrinthodont, *Proterogyrinus scheelei*, from the Lower Carboniferous. Kirtlandia 10: 1—16.
94. Ruta M, Clack JA (2006) A review of *Silvanerpeton miripedes*, a stem amniote from the Lower Carboniferous of East Kirkton, West Lothian, Scotland. Earth and Environmental Science Transactions of the Royal Society of Edinburgh 97: 31—63.
95. Snitting D (2008) A redescription of the anatomy of the Late Devonian *Spodichthys buetleri* Jarvik, 1985 (Sarcopterygii, Tetrapodomorpha) from East Greenland. Journal of Vertebrate Paleontology 28: 637—655.
96. Shubin NH, Daeschler EB, Jenkins Jr FA (2006) The pectoral fin of *Tiktaalik roseae* and the origin of the tetrapod limb. Nature 440: 764—771.
97. Downs JP, Daeschler EB, Jenkins Jr FA, Shubin NH (2008) The cranial endoskeleton of *Tiktaalik roseae*. Nature 455: 925—929.
98. Snitting D (2008) Anatomy of *Tristichopterus*, with comments on the validity of *Eusthenopteron*. Paper III. Morphology, Taxonomy, and Interrelationships of tristichopterid fishes (Sarcopterygii, Tetrapodomorpha) PhD Thesis, Subdepartment of Evolutionary Organismal Biology, Uppsala University, Uppsala.
99. Egerton PG (1861) *Tristichopterus alatus*. Memoirs of the Geological Survey of the UK, Figures and Descriptions Illustrative of British Organic Remains 10: 51—55.
100. Ahlberg P, Lukševičs E, Lebedev O (1994) The first tetrapod finds from the Devonian (Upper Famennian) of Latvia. Philosophical Transactions: Biological Sciences 343: 303—328.
101. Lombard RE, Bolt JR (1995) A new primitive tetrapod, *Whatcheeria deltae*, from the Lower Carboniferous of Iowa. Palaeontology 38: 471—494.
102. Lombard RE, Bolt JR (2006) The mandible of *Whatcheeria deltae*, an early tetrapod from the Late Mississippian of Iowa. In: Carrano MT, Blob, R.W., Gaudin, T.J. and Wible, J.R., editor. Amniote Paleobiology: Perspectives on the Evolution of Mammals, Birds, and Reptiles. Chicago: University of Chicago Press. pp. 21—52.
103. Chang M-m (1982) The braincase of *Youngolepis*, a Lower Devonian crossopterygian from Yunnan, south-western China. Stockholm: University of Stockholm, and Section of Palaeozoology, Swedish Museum of Natural History.
104. Chang M-m (1991) Head exoskeleton and shoulder girdle of *Youngolepis*. In: Chang M-m, Liu, Y.H. and Zhang, G.R., editor. Early Vertebrates and Related Problems of Evolutionary Biology. Beijing: Science Press. pp. 355—378.
105. Chang M-m (2004) Synapomorphies and scenarios—more characters of *Youngolepis* betraying its affinity to the Dipnoi. In: Arratia G, Wilson, M.V.H. and Cloutier, R., editor. Recent Advances in the Origin and Early Radiation of Vertebrates. München: Verlag Dr. Friedrich Pfeil. pp. 665—686.

106. Chang M-m, Smith MM (1992) Is *Youngolepis* a Porolepiform? Journal of Vertebrate Paleontology 12: 294—312.
107. Swofford D (2002) PAUP: phylogenetic analysis using parsimony, version 4.0 b10. Sunderland.
108. Huelsenbeck JP, Ronquist, F., Nielsen, R. and Bollback, J.P. (2001) Bayesian inference of phylogeny and its impact on evolutionary biology. Science 294: 2310—2314.
109. Ronquist F, Huelsenbeck JP (2003) MrBayes 3: Bayesian phylogenetic inference under mixed models. Bioinformatics 19: 1572—1574.
110. Nixon KC (1999) The Parsimony Ratchet, a new method for rapid parsimony analysis. Cladistics 15: 407-414.
111. Goloboff PA (1999) Analyzing large data sets in reasonable times: solutions for composite optima. Cladistics 15: 415—428.
112. Maddison DR, Maddison WP (2000) MacClade: Analysis of Phylogeny and Character Evolution. 4.0 ed. Sunderland, Massachusetts: Sinauer Associates.
