# Supplementary figures and images for: A Marine Stem-Tetrapod from the Devonian of Western North America
Source: PLoS One. 2012 Mar 20;7(3):e33683. doi: 10.1371/journal.pone.0033683 (PMC3308997; doi:10.1371/journal.pone.0033683)

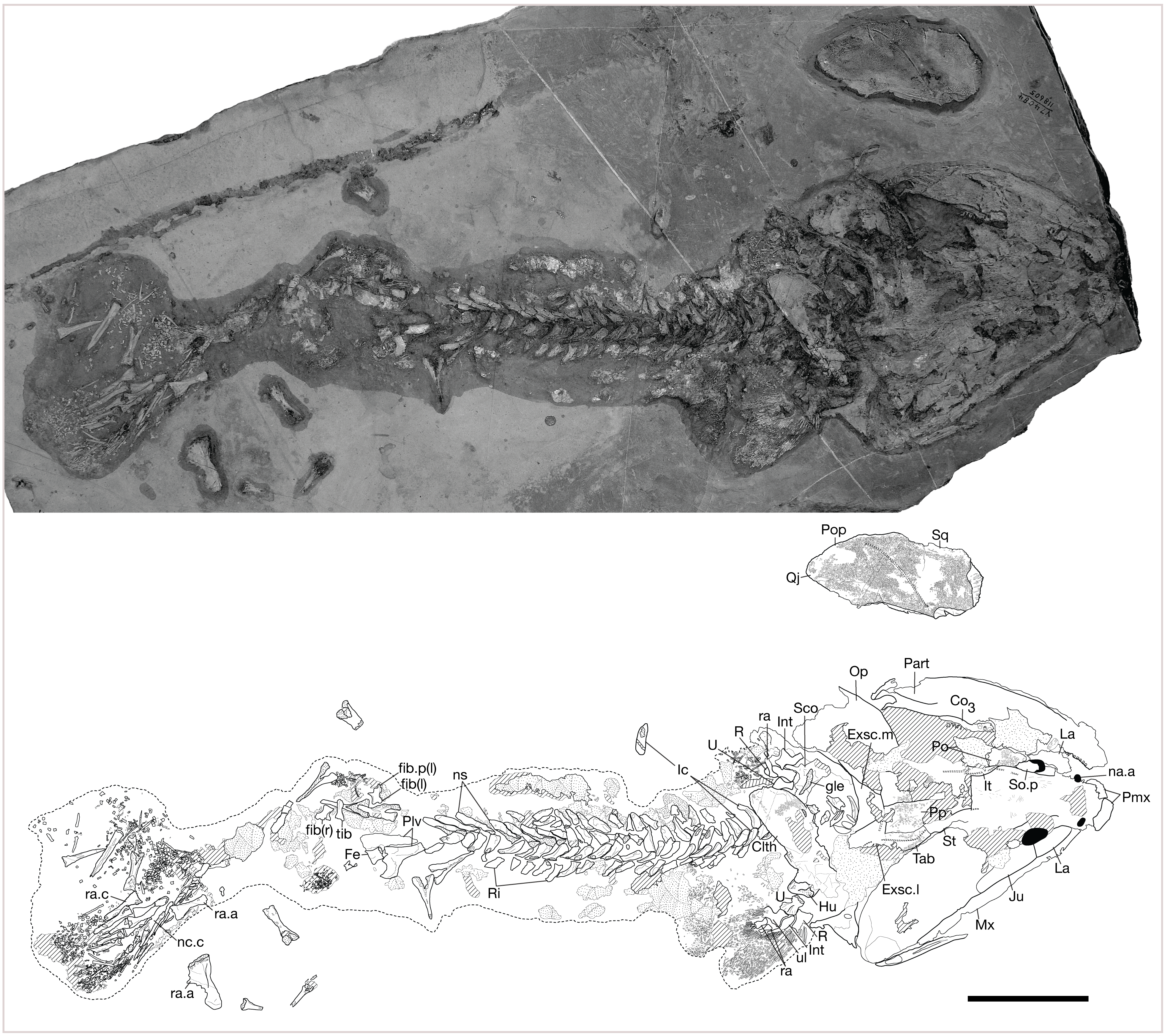

Supplement: Figure S1 — Close-up of UCMP 118605 and specimen drawing. UCMP 118605, holotype, in dorsal, lateral and ventral view. See main text for details; right is anterior. Abbreviations: ba.a, anal basal; Clth, cleithrum; Clv, clavicle; Co3, posterior coronoid; Exsc.l, lateral extrascapular; Exsc.m, median extrascapular; Fe, femur; fib, fibula; fib.p, posterior process of the fibula; gle, glenoid fossa; Hu, humerus; Ic, intercentrum; Int, intermedium; It, intertemporal; Ju, jugal; La, lacrimal; Mx, maxilla; na.a, anterior naris; nc.c, notochordal canal; ns, neural spine; Part, prearticular; Plv, pelvis; Pmx, premaxilla; Po, postorbital; Pop, preopercular; Pp, postparietal; Qj, quadratojugal; R, radius; ra, radial; ra.a, anal radial; ra.c, caudal radial; Ri, rib; Sca, scale; Sco, scapulocoracoid; St, supratemporal; So.p, posterior supraorbital; Sq, squamosal; Tab, tabular; tib, tibia; U, ulna; ul, ulnare; Scale bar equals 10 cm. (TIF) [file pone.0033683.s002.tif]

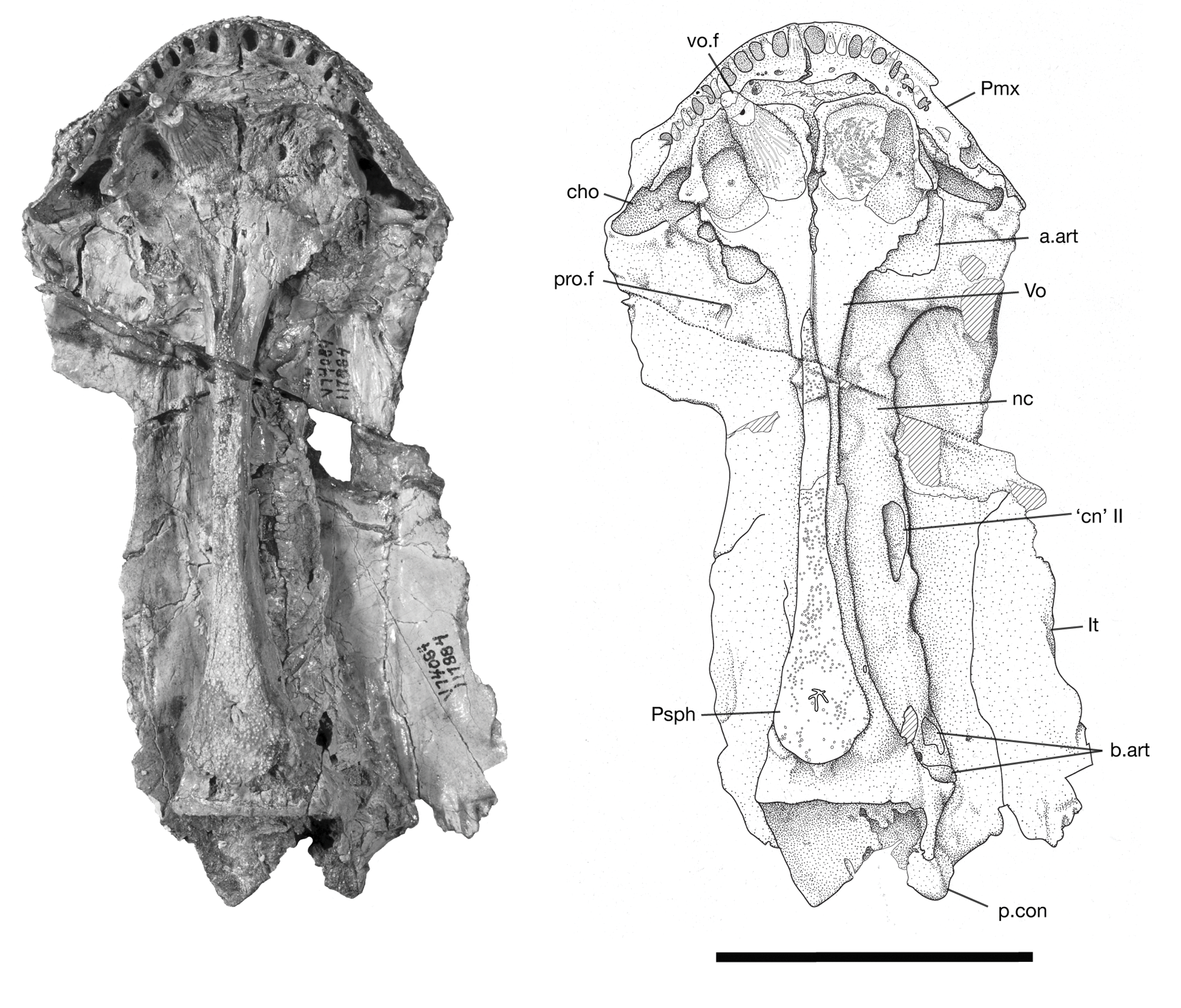

Supplement: Figure S2 — Ethmoid palatal region and interpretive drawing of UCMP 117884. Anterior is toward the top of the page. Abbreviations: a.art, autopalatine articulation; b.art, basal articulation of basipterygoid process; cho, choana; ‘cn’ II, optic nerve; It, intertemporal, nc, neurocranium; p.con, processes connectens; Pmx, premaxilla; pro.f, profundus foramen; Psph, parasphenoid; Vo, vomer; vo.f, vomerine fang. ‘CN’ is in scare quotes because the optic nerve is not a real cranial nerve but a special-sensory extension of the diencephalon. Scale bar equals 5 cm. (TIF) [file pone.0033683.s003.tif]

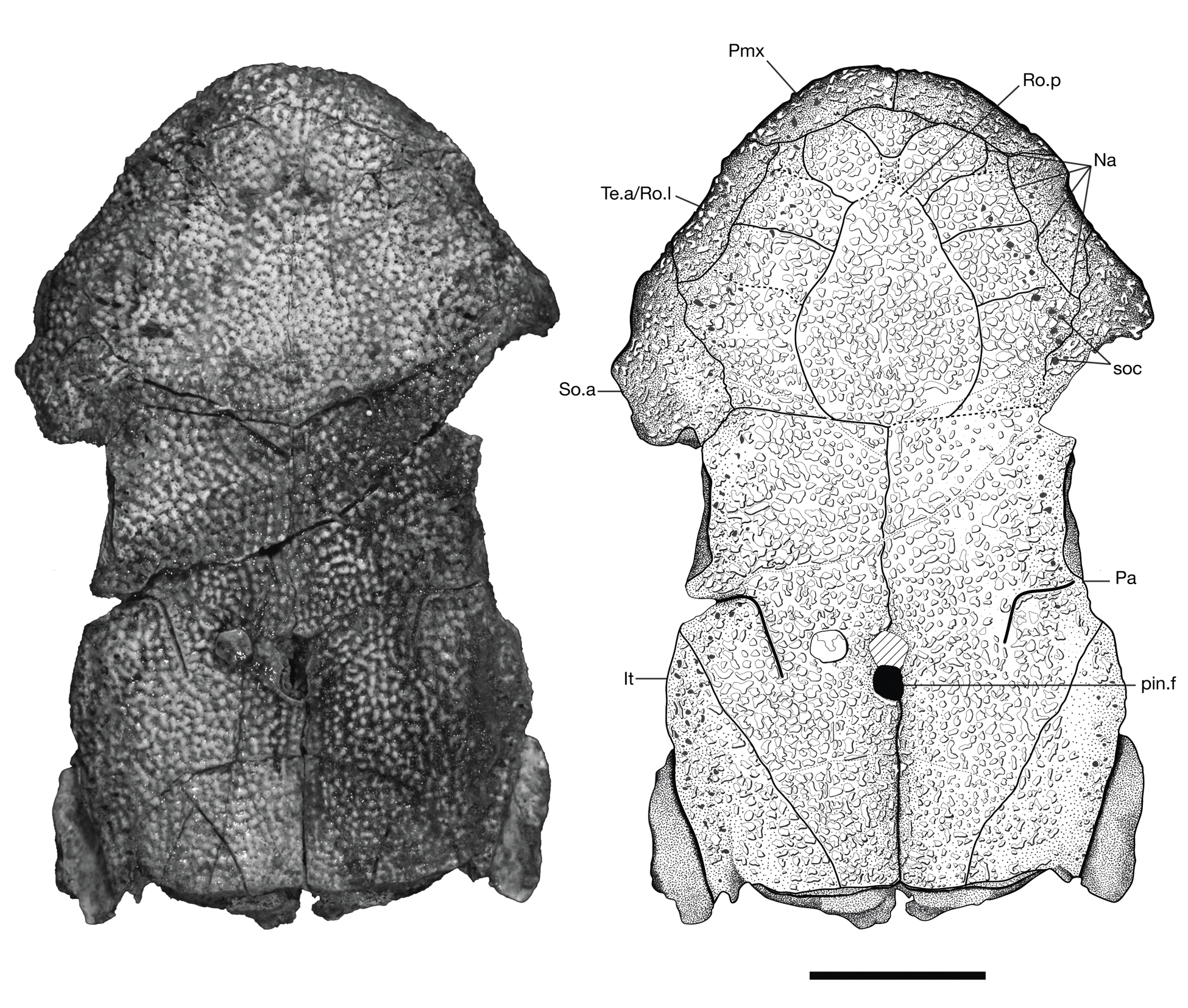

Supplement: Figure S3 — Ethmoid skull roof and interpretive drawing of juvenile specimen UCMP 118283. Aside from the fusion of the anterior tectal and lateral rostral (similar to the adult specimen, UCMP 117884), many of the remaining roofing bones are unfused. The snout of this specimen is also proportionally shorter than the adult (when pineal foramina are aligned), suggesting substantial allometric change during ontogeny. In addition, it lacks the recessed tongue-and-groove articulations spanning the dermal intracranial joint, suggesting acquisition later in life. Anterior is toward the top of the page. Abbreviations: It, intertemporal; Na, nasal; Pa, parietal; pin.f, pineal foramen; Pmx, premaxilla; Ro.p, median postrostral; So.a, anterior supraorbital; soc, supraorbital canal; Te.a/Ro.l, (fused) anterior tectal/lateral rostral. Scale bar equals 5 mm. (TIF) [file pone.0033683.s004.tif]

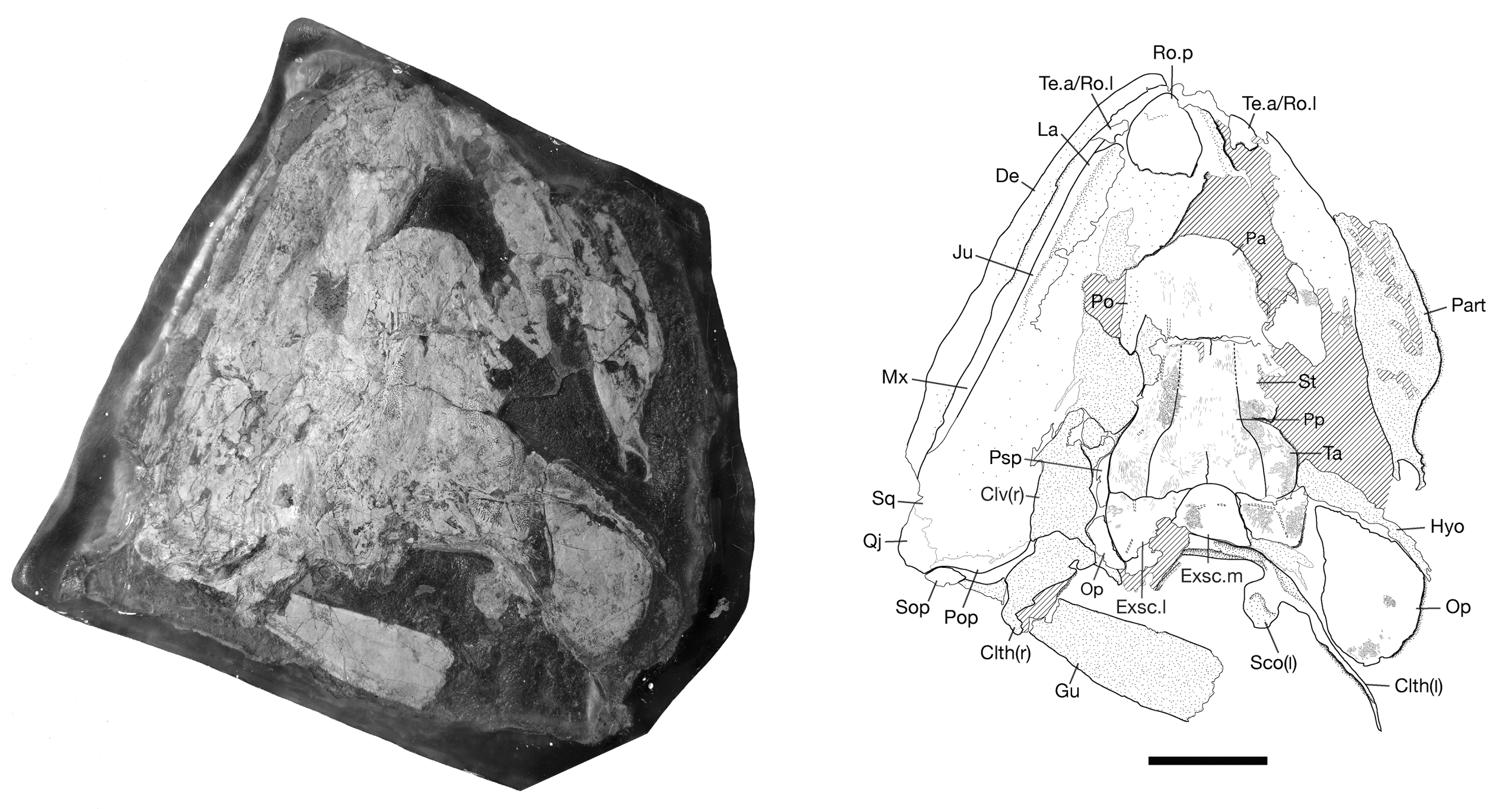

Supplement: Figure S4 — Skull, partial shoulder, and interpretive drawing of UCMP 190999. Anterior is toward the top of the page. Abbreviations: Clth, cleithrum; Clv, clavicle; De, dentary; Exsc.l, lateral extrascapular; Exsc.m, median extrascapular; Gu, lateral gular; Hyo, hyomandibular; Ju, jugal; La, lacrimal; Mx, maxilla; Op, operculum; Pa, parietal; Part, prearticular; Pop, preoperculum; Pp, postparietal; Psp, postspiracular; Qj, quadratojugal; Ro.p, median postrostral; Sco, scapulocoracoid; Sop, suboperculum; Sq, squamosal; St, supratemporal; Ta, tabular; Te.a/Ro.l, (fused) anterior tectal/lateral rostral. (l) or (r) refers to left or right when displaced from natural side. Scale bar equals 5 cm. (TIF) [file pone.0033683.s005.tif]

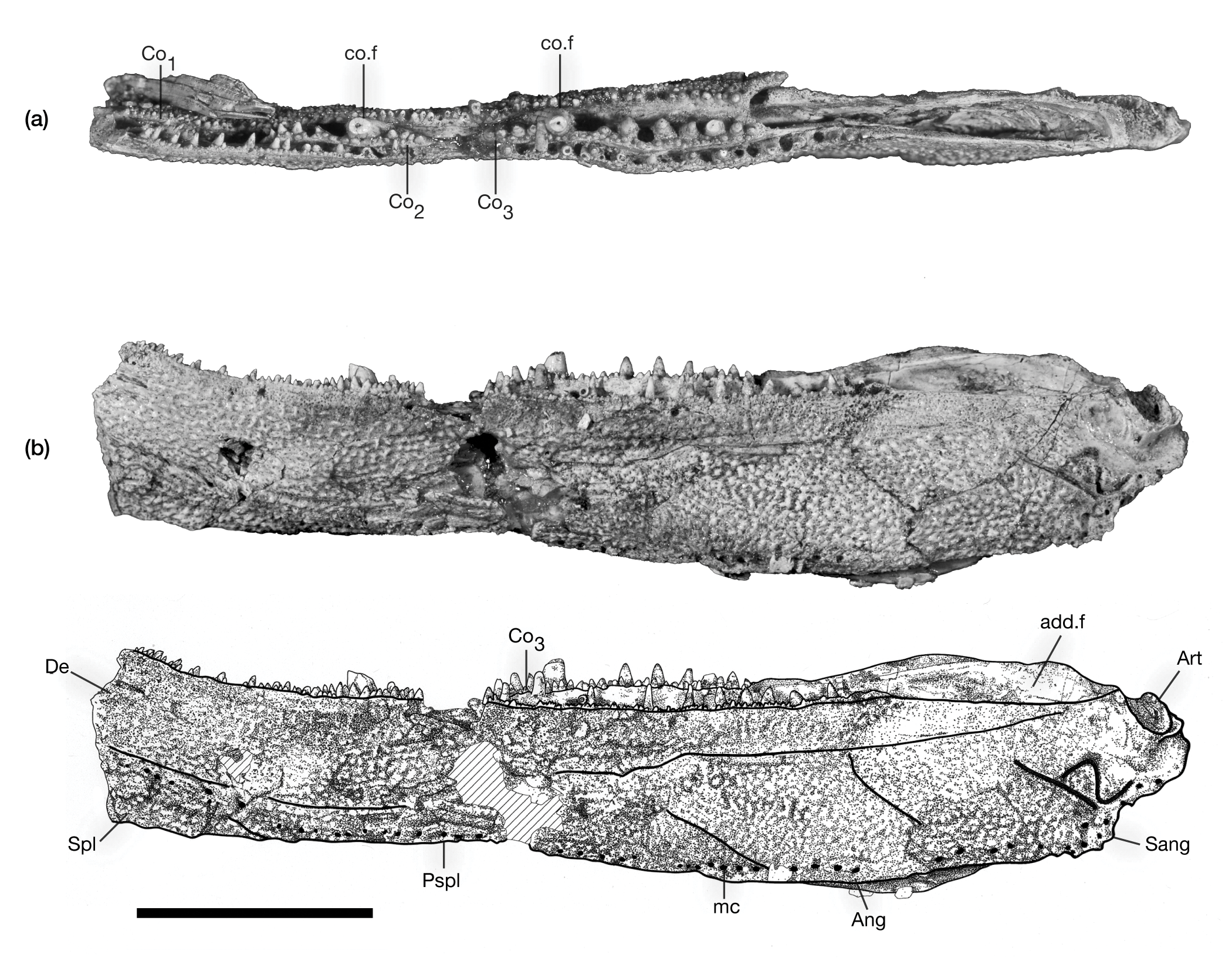

Supplement: Figure S5 — Lower Jaw of UCMP 123135. (a) Dorsal view; (b) lateral view and interpretive drawing. Left is anterior. Abbreviations: add.f, adductor fossa; Ang, angular; Art, articular; Co1, anterior coronoid; Co2, middle coronoid; Co3, posterior coronoid; co.f, coronoid fang; De, dentary; mc, mandibular canal; Pspl, postsplenial; Sang, surangular; Spl, splenial. Scale bar equals 10 mm. (TIF) [file pone.0033683.s006.tif]

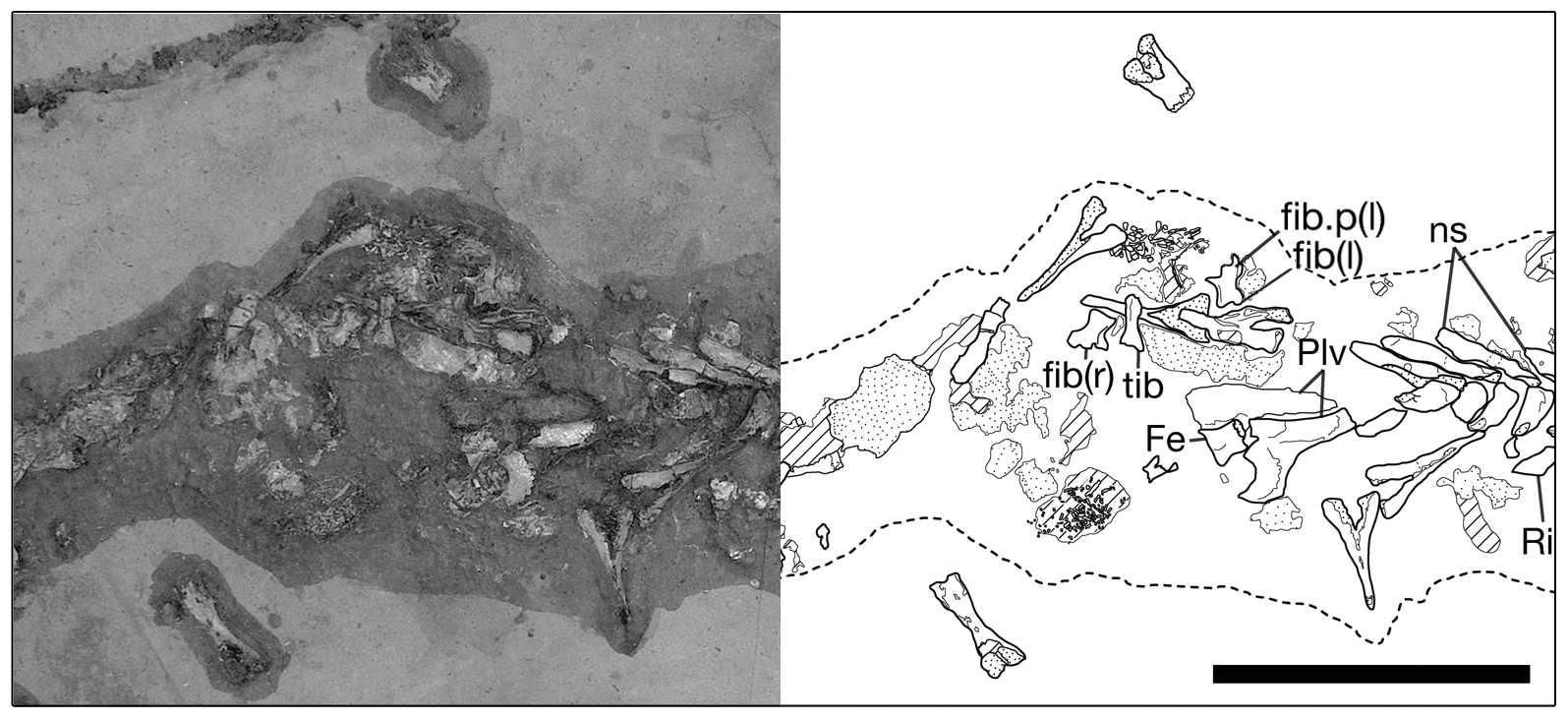

Supplement: Figure S6 — Close-up of the pelvic region of UCMP 118605 highlighting the reduced postaxial fibular processes. Abbreviations: fe, femur; fib, fibula; Plv, pelvis; ns, neural spine; Ri, rib. (l) or (r) refers to left or right. Scale bar equals 10 cm. (TIF) [file pone.0033683.s007.tif]

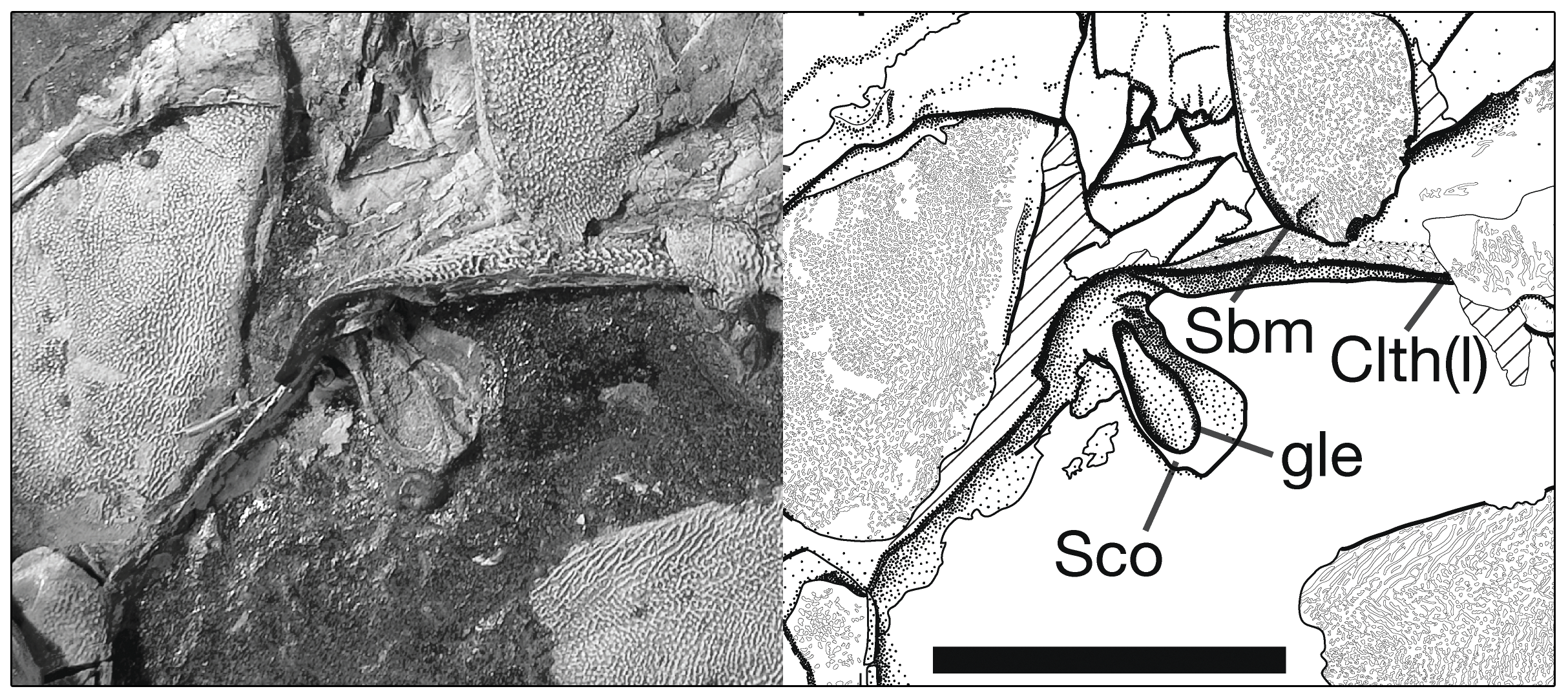

Supplement: Figure S7 — Close-up of the elongate glenoid fossa of UCMP 190999. Abbreviations: Clth, cleithrum; gle, glenoid fossa; Sbm, submandibular; Sco, scapulocoracoid. (l) refers to left. Scale bar equals 5 cm. (TIF) [file pone.0033683.s008.tif]
